# A new species of fringed Forest Gecko, genus *Luperosaurus* (Squamata: Gekkonidae), from Sibuyan Island, Central Philippines

**DOI:** 10.7717/peerj.20504

**Published:** 2026-03-04

**Authors:** Camila G. Meneses, Rafe M. Brown

**Affiliations:** Department of Ecology and Evolutionary Biology & Biodiversity Institute, University of Kansas, Lawrence, KS, United States of America

**Keywords:** Biodiversity, Rare species of flap-legged geckos, Wolf geckos, Taxonomy, Systematics, Oceanic island, Endemic

## Abstract

We describe a new species of *Luperosaurus* based on two specimens collected on Sibuyan Island, Romblon Province, central Philippines. The new species is phenotypically similar to *L. cumingii* (southern Luzon), *L. angliit* (northern Luzon), *L. corfieldi* (from Panay and Negros islands), and *L. macgregori* (the Babuyan and Batanes island groups), but differs from these closely related congeners and all other known *Luperosaurus* by a combination of discrete morphological characters. Extensive molecular divergence from all closely related species for which genetic data are available supports the new species as a distinct lineage. Its distribution is geographically isolated from congeners, restricted to a permanently isolated deep-water island. The new species’ extremely limited geographic range contributes to the recognition of the remaining forests of the central Philippine Romblon Island Group as a fundamental conservation priority for the archipelago.

## Introduction

The poorly known Southeast Asian and Southwest Pacific lizard genus *Luperosaurus*, known popularly as fringed geckos, wolf geckos, or flap-legged geckos, has historically been characterized by a combination of morphological features, including cutaneous expansions on the margins of the limbs, broadly dilated digits, extensive interdigital webbing, and absence of enlarged postmentals, chin shields, and differentiated subcaudals (Brown & Alcala, 1978; Brown, Supriatna & Ota, 2000; Brown, Diesmos & Duya, 2007; Brown, Diesmos & Oliveros, 2011). Since Gray originally described *L*. *cumingii* (Gray, 1845), researchers have described seven additional Philippine *Luperosaurus* species, six of which remain recognized within *Luperosaurus*: *L*. *angliit*, *L*. *corfieldi*, *L*. *joloensis*, *L. kubli*, *L. macgregori*, and *L*. *palawanensis* (Taylor, 1918; Taylor, 1922; Brown & Alcala, 1978; Brown & Diesmos, 2000; Gaulke, Rösler & Brown, 2007; Brown, Diesmos & Duya, 2007; Brown, Diesmos & Oliveros, 2011; Brown et al., 2012). Taxonomists transferred, the southern Palawan high-elevation *L*. *gulat*, to the genus *Gekko*, subgenus *Balawangekko* (Brown, Diesmos & Oliveros, 2011; Brown et al., 2011; Wood Jr et al., 2020). Additionally, Oliver et al. (2018) proposed reclassifying *Luperosaurus joloensis* as a species of *Lepidodactylus*, reflecting unresolved instability of differing phylogenetic placement of species currently assigned to these two genera (Brown et al., 2012; Oliver et al., 2018; Wood Jr et al., 2020) and a general expectation of additional, ongoing revisionary work within these Philippine gekkonid lizards.

As of a decade ago, the relationship of *Luperosaurus* to other gekkonine genera (*Gekko, Gehyra, Hemiphyllodactylus, Lepidodactylus, Perochirus, Pseudogekko,* and *Ptychozoon*) remained uncertain (Russell, 1979; Ota, Sengoku & Hikida, 1996; Brown, Supriatna & Ota, 2000; Russell & Bauer, 2002). However, molecular phylogenetic analyses have demonstrated the paraphyly of *Gekko* and *Lepidodactylus* with respect to *Ptychozoon*, *Pseudogekko*, and several species currently assigned to *Luperosaurus* (*L*. *angliit, L*. *cumingii, L*. *macgregori*, and *L*. *joloensis*; Brown et al., 2012; Heinicke et al., 2012; Oliver et al., 2018; Wood Jr et al., 2020).

Outside of the archipelago, five non-Philippine species formerly assigned to *Luperosaurus* are currently recognized as: *Gekko browni* (Malay Peninsula and northern Borneo; Russell, 1979; Wood Jr et al., 2020), *Gekko brooksii* (Sumatra; Russell, 1979; Wood Jr et al., 2020), *Luperosaurus yasumai* (Borneo; Ota, Sengoku & Hikida, 1996), *Luperosaurus sorok* (Borneo; Das, Lakim & Kandaung, 2008; Wood Jr et al., 2020; Fukuyama, Hossman & Nishikawa, 2022), and *Gekko iskandari* (Sulawesi; Brown, Supriatna & Ota, 2000; Wood Jr et al., 2020). Two additional nominal species have been synonymized following taxonomic revisions: *Luperosaurus amissus* (Taylor, 1962), placed in the synonymy of *Gekko hokouensis* by Brown & Alcala (1978) (see also Ota et al., 1989), and *Luperosaurus serraticaudus* (Ota et al., 1989; Brown, Supriatna & Ota, 2000), now considered a junior synonym of *Gekko browni* Brown, Supriatna & Ota, 2000; Wood Jr et al., 2020).

Ota, Sengoku & Hikida (1996) originally distinguished stout-bodied species of *Luperosaurus* from the more slender-bodied taxa. Phylogenetic analyses of both morphological (Brown, Supriatna & Ota, 2000) and molecular sequence data (Brown et al., 2012; Heinicke et al., 2012) have supported this phenotypic distinction. These studies have also shown that the two phenotypic classes fall into separate clades, supporting their status as distinct evolutionary lineages. The true *Luperosaurus*, or the stout-bodied clade, includes *L*. *angliit, L*. *cumingii* (the type species for the genus), *L*. *macgregori*, *L*. *corfieldi, L*. *kubli*, *L*. *palawanensis*, and provisionally *L*. *sorok*. Because the generic assignment of *L. sorok* remains unsettled—with some recent studies and databases placing it in *Gekko* (Wood Jr et al., 2020) and others recovering it within the stout-bodied *Luperosaurus* clade (Fukuyama, Hossman & Nishikawa, 2022)—we treat it here provisionally as part of the stout-bodied group, pending further molecular sampling and taxonomic resolution. The unrelated, slender-bodied species clade includes *G*. *iskandari, G*. *browni*, and, presumably, *G*. *brooksii*, which is more closely related to *Gekko vitattus* (Brown et al., 2012; Wood Jr et al., 2020).

**Figure 1:**
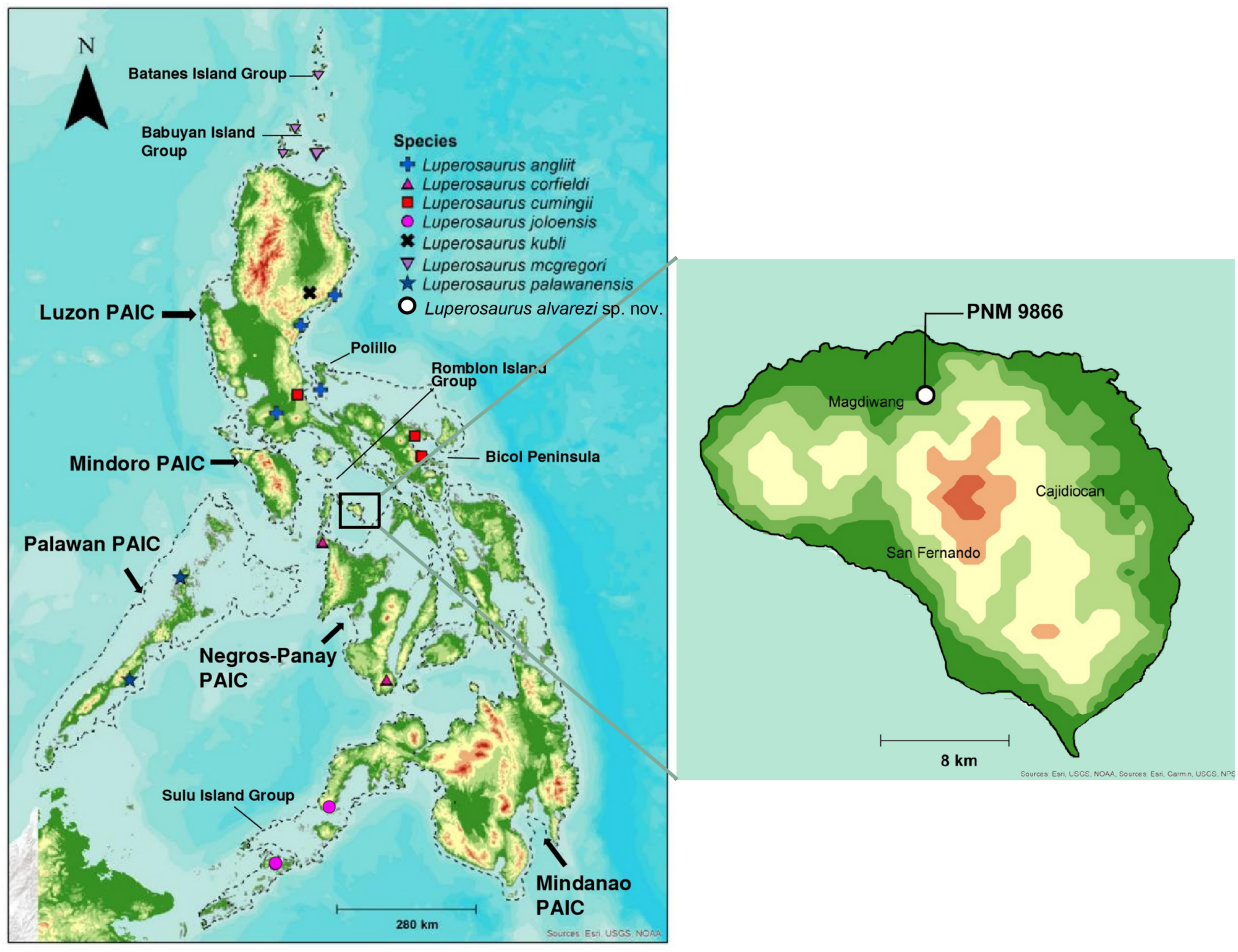
Geographic distribution of the described Philippine Luperosaurus species, with an inset map of Sibuyan Island indicating the collection site of the new species. The left panel is the map of the distribution of species of the genus Luperosaurus in the Philippines with outlines of Pleistocene Aggregate Island Complexes (PAICs) indicated (light gray) by tracing 120 m bathymetric contours. Labeled islands are discussed in the text. The right panel is a map of Sibuyan Island, with the type locality of *Luperosaurus alvarezi* sp. nov. (municipality of Magdiwang, Mt. Guiting-Guiting) indicated with a white circle (Holotype: PNM 9866).

During a herpetological inventory of Sibuyan Island, Romblon Province, central Philippines, one of us (CGM) collected a putative new species of *Luperosaurus* from low-elevation forests of Mt. Guiting-Guiting Natural Park, where previous inventory work had failed to detect the species (Brown & Alcala, 1970; Brown & Alcala, 1978; Siler et al., 2012). The new species possesses a blend of character states, intermediate between southern Luzon Island’s *L*. *cumingii* (Brown, Diesmos & Oliveros, 2011; characterized by extensive cutaneous expansions and ornate tubercles bordering the limbs and tail) and the West Visayan *L. corfieldi* (Gaulke, Rösler & Brown, 2007; Dolino et al., 2009; a much smoother-bodied, non-tuberculate species, lacking conspicuous dermal expansions).

In this paper, we describe a new species corresponding to the Sibuyan Island population of *Luperosaurus*. We use a combination of body size and shape and meristic data of external morphology (scale counts) to demonstrate the distinctive nature of this isolated insular lineage and use molecular sequence data to investigate its affinities. Our analysis demonstrates strongly supported phylogenetic affinities to populations from Luzon, including the type species of the genus, *L*. *cumingii* (southern Luzon); *L*. *angliit* (northern Luzon and the Babuyan islands); *L*. *macgregori* (the Babuyan island groups); and presumably the West Visayan endemic *L*. *corfieldi* (from Panay and Negros; Brown et al., 2012; Oliver et al., 2018; Fig. 1), for which no tissue samples are available. Even without molecular data from *L*. *corfieldi*, we find the degree of phenotypic distinctiveness (multiple apparently fixed phenotypic character state differences that preclude assignment of the Sibuyan population to either *L*. *corfieldi* or *L. cumingii*) to be compelling justification for the recognition of this isolated population as a full species taxon. This work aims to expand taxonomic understanding of Philippine *Luperosaurus*, contributes to our ongoing effort to clarify phylogenetic relationships within the genus and contributes to our knowledge of endemic lizard diversity on Sibuyan Island and, more broadly, within the biogeographically distinctive Romblon Island Group.

## Materials and Methods

### Ethics statement

Fieldwork and specimen collection were conducted under a Memorandum of Understanding between the University of Kansas, the University of the Philippines Los Baños (UPLB), and the UPLB-Museum of Natural History; Wildlife Gratuitous Permits to Collect Nos. MIMAROPA-2017-0005, issued by the Department of Environment and Natural Resources (DENR) of the Philippines. All procedures involving live animals were reviewed and approved by the University of Kansas Institutional Animal Care and Use Committee (IACUC Approval No. 158-04) and followed internationally accepted standards for the ethical treatment of animals in field research. Field activities were carried out in close coordination with local government units (LGUs) and community stakeholders to ensure adherence to local conservation policies and ethical guidelines. The electronic version of this article in Portable Document Format (PDF) will represent a published work according to the International Commission on Zoological Nomenclature (ICZN), and hence the new names contained in the electronic version are effectively published under that Code from the electronic edition alone. This published work and the nomenclatural acts it contains have been registered in ZooBank, the online registration system for the ICZN. The ZooBank LSIDs (Life Science Identifiers) can be resolved and the associated information viewed through any standard web browser by appending the LSID to the prefix http://zoobank.org/. The LSID for this publication is: urn:lsid:zoobank.org:pub:90E18669-18E2-4EB8-A10A-3FC1591F2B87. The online version of this work is archived and available from the following digital repositories: PeerJ, PubMed Central SCIE and CLOCKSS.

**Figure 2:**
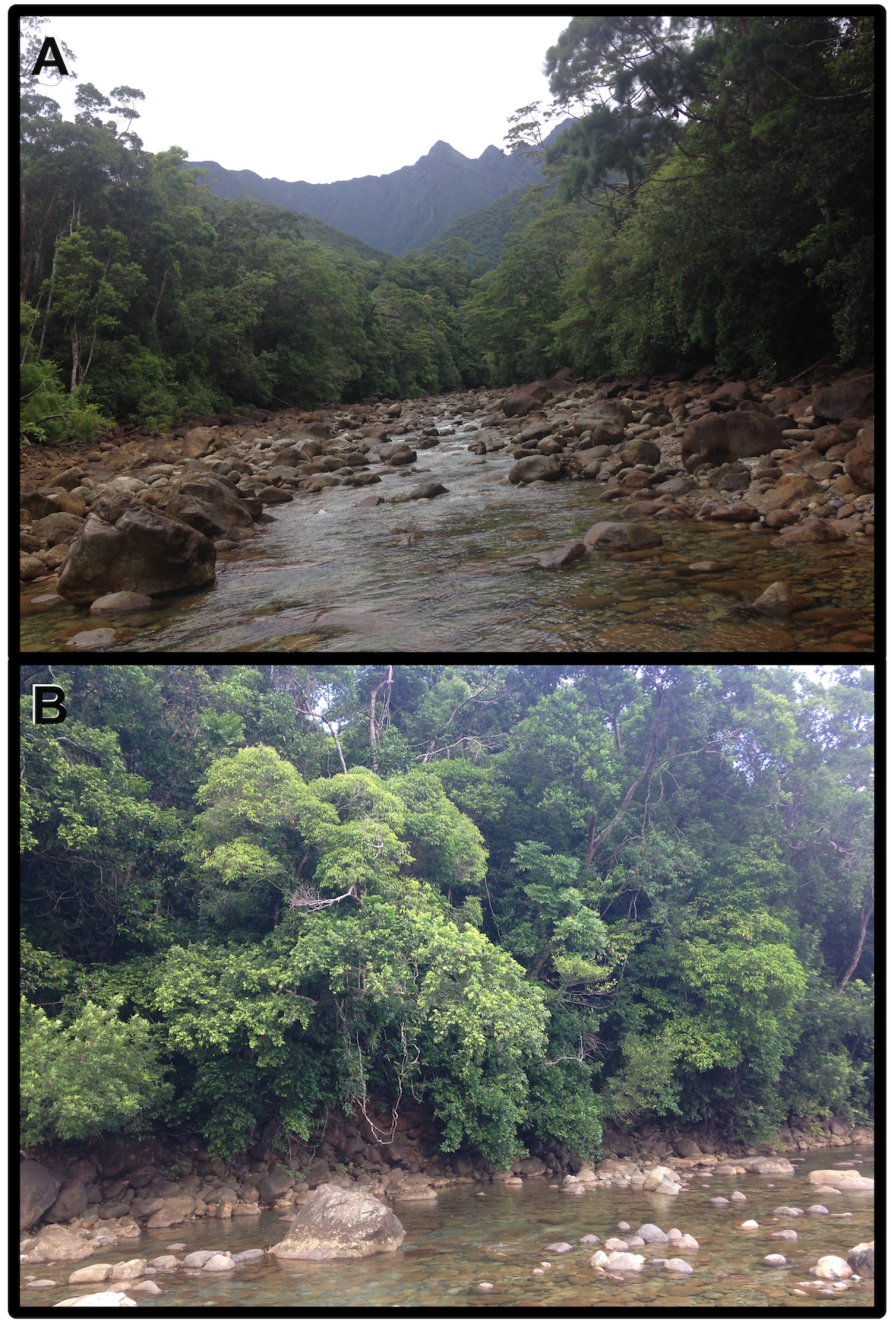
Microhabitat of *Luperosaurus alvarezi* sp. nov. on Sibuyan Island, Philippines. (A) Habitat characteristics of the type locality of *Luperosaurus alvarezi* sp. nov. at Mt. Guiting-Guiting Natural Park, Sibuyan Island, Philippines, and (B) Appearance of the microhabitat of the new species of Fringed Forest Gecko, *Luperosaurus alvarezi* sp. nov., on the Gaong River. Photographs were taken by Camila G. Meneses.

### Sampling

Two individuals of the putative new species were collected in May–June 2017 on the Gaong River, in Mt. Guiting-Guiting Natural Park, Barangay Tampayan, Sitio Logdeck, Municipality of Magdiwang, Romblon Province, Sibuyan Island, Philippines (12°28′11.5″N, 122°32′58.1″E; 48–51 m a.s.l.; Fig. 2). We collected geckos using opportunistic search methods simultaneously across multiple teams in upstream and downstream habitats. Our sampling procedure followed elevational transects at elevations ranging from 10 to 1,557 m (Goodman, Willard & Gonzales, 1995) and employed diverse sampling techniques (Heyer et al., 1994). Specimens were euthanized and fixed in 10% buffered formalin and preserved in 70% ethanol. Tissue samples were collected before fixation, stored in 100% ethanol, and deposited at the National Museum of the Philippines (PNM) and University of the Philippines Los Baños Museum of Natural History (UPLB-MNH).

### Morphological data

Measurements, following definitions in Brown et al. (2000), Brown et al. (2007), were taken to the nearest 0.1 mm with digital calipers. Sex and maturity were determined by scoring prominent secondary sexual characteristics (Brown, Supriatna & Ota, 2000; Brown, Diesmos & Duya, 2007; Brown et al., 2011; Gaulke, Rösler & Brown, 2007), presence of mature eggs in females, or hemipenes in males. Both authors examined the specimens and identified remarkably similar values for morphological characters (Table 1; Appendix S1). However, to avoid inter-observer bias (Hayek, Heyer & Gascon, 2001; Lee, 1990; Gaulke, Rösler & Brown, 2007), only data scored by CGM are presented. Mensural data include snout–vent length, tail length, axilla–groin distance, head depth, head width, head length, snout length, internarial distance, interorbital distance (anterior corner of the orbits), eye–tympanum distance, eye diameter, and auricular opening diameter, tail base height and width, femur length, tibia length, Toe I length, and Toe IV length. Meristic characters scored by both authors included scale counts: paravertebrals, longitudinal midventrals between midpoints of limb insertions, transverse midventrals (numbers of enlarged, imbricate ventral rows), total midbody scales, caudals, subcaudals, tail whorls, supralabials, infralabials, circumorbitals, Fingers I and IV scansors, Toe I and IV scansors, and numbers of pore-bearing precloacals. Additional traits documented include superciliaries, eye color, internasal contacting rostral, scales contacting nostril, subrictal tubercles, extent of web between digits III and IV, penultimate scansors, dorsal and ventrolateral body tubercles, lateral tail tubercles, anteriormost chin scales, and expansions of anterior and posterior forelimbs and hindlimbs (Brown, Diesmos & Oliveros, 2011).

**Table 1:** Distribution of selected diagnostic characters (+, present; –absent) in *Luperosaurus alvarezi* sp. nov. and the remaining Philippine species of *Luperosaurus*. Characters marked with an asterisk (*) indicate diagnostic features of *L. alvarezi* sp. nov. See (Brown et al. (2007), Brown et al. (2011): Table 1 and original descriptions for distribution of character states distinguishing the morphologically similar group of Philippine species from the remaining, phenotypically divergent non-Philippine species (and *L*. *yasumai*). Bilaterally symmetrical characters are presented for the right side of all specimens. Measurements are presented in mm and all specimens (with the exception of the *L*. *joloensis* paratype) were mature adults; the number of specimens examined of each sex (m = male, f = female, s = subadult) is indicated. Species previously referred to *Luperosaurus* but recently transferred to *Gekko (L*. *iskandari, L*. *browni, L. brooksi, L*. *sorok,* and *L*. *gulat*) are excluded from consideration here (Wood Jr et al., 2020). Sources of data (all confirmed by examination of specimens) include: (1) this study, (2) Brown et al. (2011), (3) Gaulke, Rösler & Brown (2007), (4) Brown, Supriatna & Ota (2000), (5) Brown, Diesmos & Duya (2007), (6) Brown & Alcala (1978), (7) Brown et al. (2010), Gaulke, Rösler & Brown (2007), (8) Jose et al. (2020), and (9) (Oliver et al., 2020).

**Taxon**	***alvarezi*** **, n. sp.**	***angliit***	***corfieldi***	***cumingii***	***macgregori***	***palawanensis***	***joloensis***	***kubli***
Sex	1m, 1f	3m, 1f	2m, 3f, 2s	7m, 8f, 4s	7m, 6f, 1s	1m,1f	1m, 1s	1m
Snout-to-vent length	66.1 (m) 78.3 (f)	62.5–64.7 (m) 59.4 (f)	44, 70.0 (m) 82.4–95.0 (f)	63.7–75.2 (m) 61–85 (f)	55.7–61.9 (m) 58.9–67.5 (f)	43.7 (m) 52.0 (f)	27.5 (m) 32.4 (s)	105.4 (m)
								
*Precloaco-femorals	22 (m) 22 (f)	17–19 (m) 17 (f)	16, 16 (m) 11–19 (f)	15–19 (m) 17–28 (f)	17–19 (m) 17–21 (f)	28 (m) 32 (f)	30 (m) 31 (f)	16 (m)
Toe I scansors	11, 12	9–10	10–14	11–14	11–13	9–11	8–9	12
Toe IV scansors	14, 16	11–13	14–20	13–16	13–16	12–13	9–13	16
*Superciliaries + circumorbitals	Bright yellow (all)	Bright yellow + gray	Yellow (all)	Yellow + gray	White (all)	Alternating brown and gold	Orange	Alternating brown and white
*Iris	Light gray	Silver	Dark tan-gray To reddish brown	Tan-gray with maroon reticulum	Copper, or orange	Silver to gold	Silver	Silver
Internasals contacting rostral	1	1–3	1–2	1–3	1–3	1–3	1	1
*Scales contacting nostril	5	5	4	5–6	5	5	5	5
*Head length/width	1.4, 1.5	1.4–1.5	1.2	1.2–1.3	1.4	1.2	1.3–1.4	1.2
Tail height/width	0.8, 0.8	0.8	0.8–1.0	0.70–0.90	0.8–0.9	0.8	0.5	0.8
Supralabials	14–15	12–15	14–16	14–17	13–17	11–13	11–13	13
Infralabials	13–14	14 –15	12–14	13–15	14–16	10–11	10–12	12
Postrictal and nuchal tubercles	–	–	–	Numerous, highly spinose	–	Few conical	Few conical	–
*Ornamental scales on margin of anterior forelimb expansion	Few, enlarged, flat to convex	–	–	Numerous, highly spinose	–	–	spinose, –	–
Extent of web between Toe III and IV	1/2	1/3–1/2	1/3–1/2	1/2–3/4	1/5–1/3	1/5–1/4	1/5–2/3	1/6–1/4
*Auricular opening	Elliptical, small, oblique	Large, circular	Large, subcircular	Oval, moderate, oblique	Oval, small, oblique	Large, subcircular	Oval, small, oblique	Oval, small, oblique
Penultimate scansors	Deeply notched	Deeply notched	Deeply notched	Deeply notched	Few, divided	Bowed	Deeply notched	Deeply notched
*Dorsal body tubercles	–	–	Few, convex	Many, strongly spinose	–	Few, spinose, recurved	Few, strongly spinose, recurved	–
*Ventrolateral body tubercles	–	–	Few, convex to conical	Few, convex to spinose	Few, convex	Few, spinose	Many, spinose	–
*Ventrolateral tail tubercles	Few, flat, scales enlarged, on caudal edge of whorl	Few, flat, scales enlarged, on caudal edge of whorl	–	Numerous, highly,spinose, along ventrolateral edge of whorl	Absent or few, flat,enlarged, on caudal edge of whorl	Numerous, highly spinose, encircling each whorl	Few, highly spinose, on caudal edge of whorl	–
Ventrals	Slightly enlarged, flat to granular, subimbricate	Slightly enlarged, slightly convex, juxtaposed	Small, granular, juxtaposed	Small, granular, juxtaposed	Small, granular, juxtaposed	Large, flat, subimbricate to juxtaposed	Small, flat, to convex, subimbricate	Large, flat, subimbricate
Midbody Scales	152, 154	162–173	140 –165	125–199	100–146	99–106	128–133	157
Paravertebrals	179, 182	191–207	218	180–267	214–261	–	–	–
*Longitudinal midventrals	108, 110	125–133	126	124–165	150–275	–	–	–
Subcaudals	146, 146	146, 146	–	108–196	122–204	–	–	–
Anteriormost chin scales	Slightly enlarged	small	small	Slightly enlarged	small	Slightly enlarged	Slightly enlarged	Slightly enlarged
Anterior forelimbs expansions	Wide flaps	Moderate flaps	Wide flaps	Wide flaps	Narrow flaps	–	Moderate flaps	–
Posterior forelimbs expansions	Wide flaps	Narrow flaps	Wide flaps	Wide flaps	Moderate flaps	–	Moderate flaps	Minute flaps
*Anterior hindlimbs expansions	Moderate flaps	Narrow flaps	Wide flaps	Narrow flaps	–	–	Narrow flaps	–
Posterior hindlimbs expansions	Wide flaps	Moderate flaps	Wide flaps	Wide flaps	Moderate flaps	Wide flaps	Wide flaps	Moderate flaps
Source	(1)	(2)	(2,3)	(1,3,4,6)	(3,5,6)	(4,6,8)	(1,4,6)	(1,5,9)

**Notes.**

a Specimens of *Luperosaurus cumingii* examined by us had 184–199 midbody scales (in three *L*. *cumingii* specimens from known localities on the Bicol Peninsula); specimens in European collections (see Gaulke, Rösler & Brown, 2007), without precise locality data (*i.e.*, ZMB 5578, “Philippines,” SMF 9044 “Central Luzon”), both have 182 midbody scale rows; one specimen (NMW 17985.1, from “Camiguin Island”) had 194 midbody scales. This latter record creates substantial uncertainty given that it is not clear whether this record originates on Camiguin Norte Island, north of Luzon (within the defined range of *L*. *angliit*; Brown et al., 2011) or Camiguin Sur Island, just north of Mindanao Island (which we find doubtful, given that no specimens of any *Luperosaurus* species have ever been verified from Mindanao Island proper, or even the Mindanao PAIC faunal region, other than *L*. *joloensis* from extreme southwestern Mindanao; Brown & Diesmos, 2000; Gaulke, Rösler & Brown, 2007). Specimens of *Luperosaurus corfieldi* examined by us had 216 paravertebral scales and 126 longitudinal midventrals obtained from CAS 182570 only; three specimens (PNM 7919, PNM 8480, and CAS 182570, from “Panay” and “Negros” Islands) had 140 and 165 midbody scales. Two female L. corfieldi (SVL = 95 mm) were released in the wild; no voucher specimens are available (Gaulke, Rösler & Brown, 2007).

### Molecular data and phylogenetic analysis

We included in our analysis previously published sequences of the mitochondrial NADH dehydrogenase subunit two gene (ND2) from *Luperosaurus* and other closely related Philippine gekkonid species (Brown et al., 2012; Meneses et al., 2019; Meneses et al., 2020; Siler et al., 2014; Wood Jr et al., 2020, see Table S1). Ingroup sampling included 11 *Luperosaurus* individuals: two newly collected specimens of the putative new species (PNM 9866 and UPLB-MNH-Z-NS 4622) from Sibuyan Island, and nine others representing both new and published ND2 sequence data. These included representatives from four of the 13 described species: *L. angliit*, *L. cumingii*, *L. joloensis*, and *L. macgregori*, which comprise the “true” *Luperosaurus* clade, defined as the monophyletic group containing the type species (Brown et al., 2012; Wood Jr et al., 2020).

We could not include *L*. *corfieldi*, *L*. *kubli*, and *L*. *palawanensis* in our analysis, because tissue samples for these taxa remain unavailable. To test species monophyly and assess taxonomic hypotheses, we included outgroup sequences from the genus *Lepidodactylus*, obtained from GenBank and selected based on prior gekkonid phylogenetic studies in the Philippines (Brown et al., 2012; Siler et al., 2014; Oliver et al., 2018; see Table S1).

Recent taxonomic revisions based on a phylogenetic hypothesis inferred from 5,472 ultraconserved element (UCE) loci placed the Palawan endemic *Luperosaurus gulat* within the genus *Gekko*, along with the Sulawesi endemics *G*. *iskandari* and *G*. *browni* (Wood Jr et al., 2020). Although no sequence data are available for *L*. *palawanensis*, previous authors have presumed its placement within this same clade. Consequently, we excluded both Palawan endemics species (*G*. *gulat* and *L*. *palawanensis*) from the Philippine *Luperosaurus* clade in our study (Brown et al., 2012; Meneses et al., 2022; Siler et al., 2014; Wood Jr et al., 2020).

We extracted genomic DNA from liver tissue samples preserved in 100% ethanol using a Maxwell^®^ 16 Tissue DNA Purification Kit and Promega Maxwell^®^ Rapid Sample Concentrator Instrument at the University of Kansas Biodiversity Institute’s Molecular Genomics Laboratory. To target the mitochondrial ND2 gene, we amplified a fragment ranging from 1,247 to 1,438 base pairs using PCR and primers developed by Macey et al. (1997) and Macey et al. (1999): forward primer Metf6 (5′–AAGCTTTCGGGCCCATACC–3′) and reverse primer CO1R1 (5′–AGRGTGCCAATGTCTTTGTGRTT–3′). Each reaction included 32 amplification cycles with an annealing temperature of 48 °C, allowing for ±1–3 °C variation. Amplified products were visualized on 1.5% agarose gels, purified, and outsource to GENEWIZ^®^ for sequencing.

We generated new ND2 sequences for nine individuals, representing three species sampled from four different localities (Table S1). Sequences were assembled de novo and manually edited in Geneious (https://www.geneious.com), following established workflows (Edgar, 2004; Kearse et al., 2012). Alignment was performed using the MAFFTplugin (Katoh & Standley, 2013) in Geneious, with default parameters.

To examine the monophyly of *Luperosaurus* and determine the placement of the RIG population, we constructed phylogenetic trees using both Maximum Likelihood (ML) and Bayesian Inference (BI) approaches. For the ML analysis, we used IQ-TREE v2.3.6 (Nguyen et al., 2015; Trifinopoulos et al., 2016), treating each codon position as an independent partition. Substitution models were selected using ModelFinder (Chernomor, von Haeseler & Minh, 2016; Kalyaanamoorthy et al., 2017). We assessed branch support using 10,000 ultrafast bootstrap replicates, and considered nodes with support values of ≥95% to be strongly supported (Minh, Nguyen & von Haeseler, 2013). The tree with the lowest Akaike Information Criterion (AIC) value was selected as the best-fit topology.

For Bayesian inference, we used BEAST v2.7.7 (Bouckaert et al., 2014), again partitioning the data by codon position. Instead of pre-selecting substitution models, we used bModelTest v1.3.3 (Bouckaert & Drummond, 2017) to explore model space during tree inference. BEAST was run with an optimized relaxed log-normal molecular clock to account for rate variation across branches and used a Yule tree prior with uniform distribution, appropriate for modeling divergence among species. We ran two independent MCMC chains, each for 10 million generations (for a combined total of 20 million), logging parameters every 1,000 generations. Log files were combined using LogCombiner, and the first 10% of generations were discarded as burn-in.

We monitored convergence using Tracer v1.7.2 (Rambaut et al., 2018), confirming that all estimated parameters had effective sample sizes (ESS) >200. The final consensus tree was summarized in TreeAnnotator v2.7.4 as a maximum clade credibility (MCC) tree with mean node heights. Nodes with posterior probabilities ≥0.95 were considered strongly supported. All trees were visualized using FigTree v1.4.4 (Rambaut et al., 2018). Newly generated ND2 gene sequences have been deposited in GenBank (see accession numbers in Table S1).

### Species concept

In this and related studies, we confirmed the status of putative evolutionary lineages by applying the General Lineage Species Concept of De Queiroz (1999), De Queiroz (2005), as a natural extension of the Evolutionary Species Concept (Simpson, 1961; Wiley, 1978). The application of lineage-based species models to diagnosable, allopatric, island-endemic species, like the one assessed here, is straightforward in island archipelagos like the Philippines. In such cases, the geological history of relevant landmasses has been so well characterized (Hall, 1996; Hall, 1998; Hall, 2001; Hall, 2002; Yumul Jr et al., 2003; Yumul et al., 2009), such that the history of population isolation on a single island or island bank (Brown & Diesmos, 2009) is uncontroversial (Brown & Guttman, 2002; Brown, 2016; Brown et al., 2013; Brown et al., 2016). As such, we recognize the Sibuyan *Luperosaurus* population as a distinct species in that it apparently satisfies two essential criteria: (1) a distinct inferred evolutionary history as an ancestor-descendant series of populations (a cohesive lineage) with (2) a predictable evolutionary fate, or future, as an evolutionary lineage with no expectation of reticulation or gene flow with other similarly isolated island endemic populations (Wiley, 1978; Frost & Hillis, 1990).

## Results

Although only two vouchered, adult specimens of the new putative species were collected, we are confident in making the current taxonomic designation on account of their shared possession of multiple discrete phenotypic character state differences and inferred phylogenetic placement as a first-diverging lineage within the Philippine *Luperosaurus* clade. The presence of fixed or discretely varying (diagnostic) characteristics of external morphology and non-overlapping ranges of character states unequivocally prevents the assignment of the Sibuyan specimens to any known species of *Luperosaurus* (Table 1). Discrete ranges in meristic data and categorical character state disparities are summarized below, contributing to both the new species’ diagnosis and comparisons with related species.

### Systematics accounts

**Table:** 

**Squamata Oppel, 1811**
**Gekkonidae Gray, 1825**
***Luperosaurus*** **Gray, 1845**

**Table:** 

**Figure 3:**
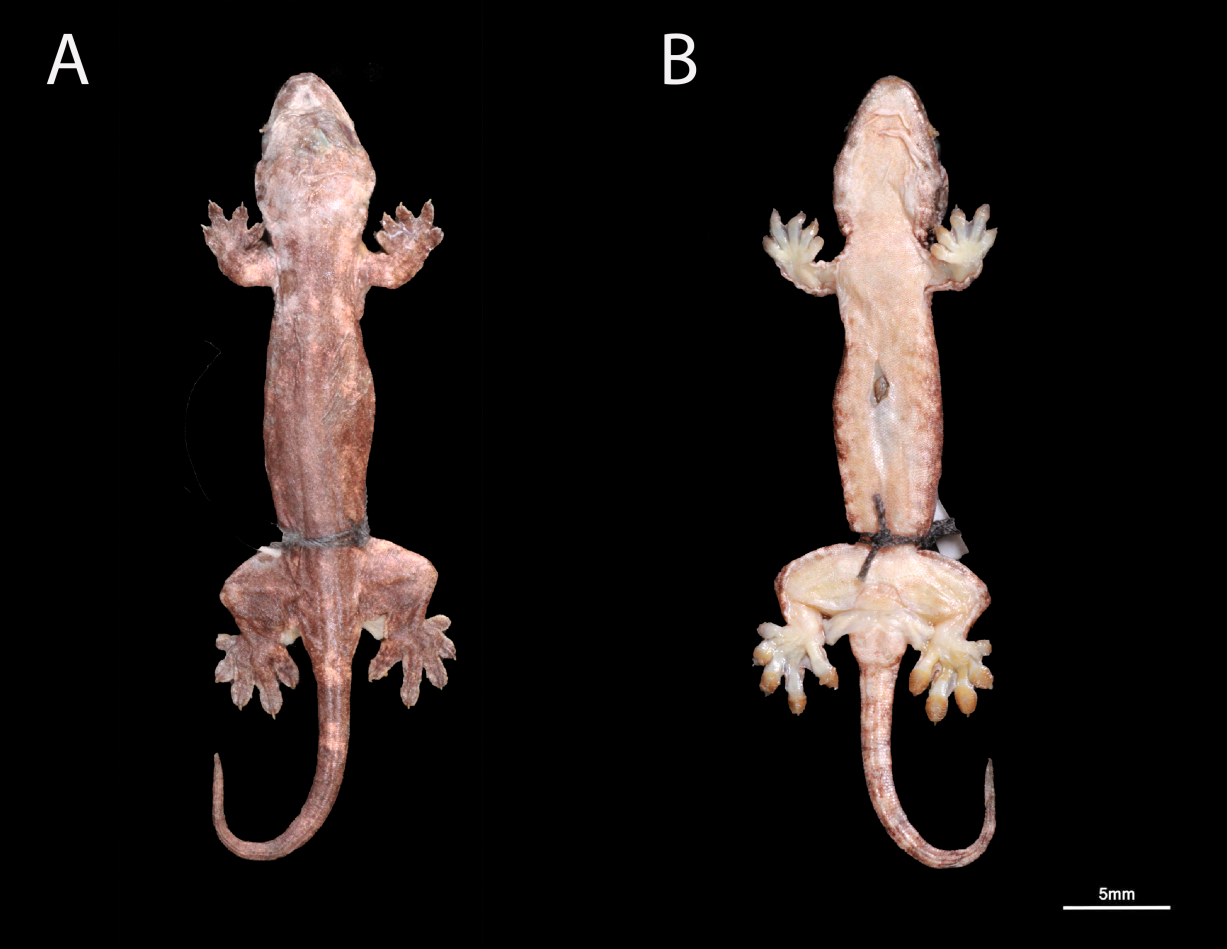
Holotype of *Luperosaurus alvarezi* sp. nov. in preservative. Dorsal (A) and ventral (B) views of the body of the holotype of *Luperosaurus alvarezi* sp. nov. (PNM 9866: adult male; SVL 66.1 mm) from Mt. Guiting-Guting Natural Park, Romblon Province, Sibuyan Island. Noted robust, stout body shape, moderate cutaneous expansions on anterior and posterior edges of forelimbs and hindlimbs and ventrolateral surfaces of the body, and tail base lacking spinose tubercles or denticulate tail lobes.

**Figure 4:**
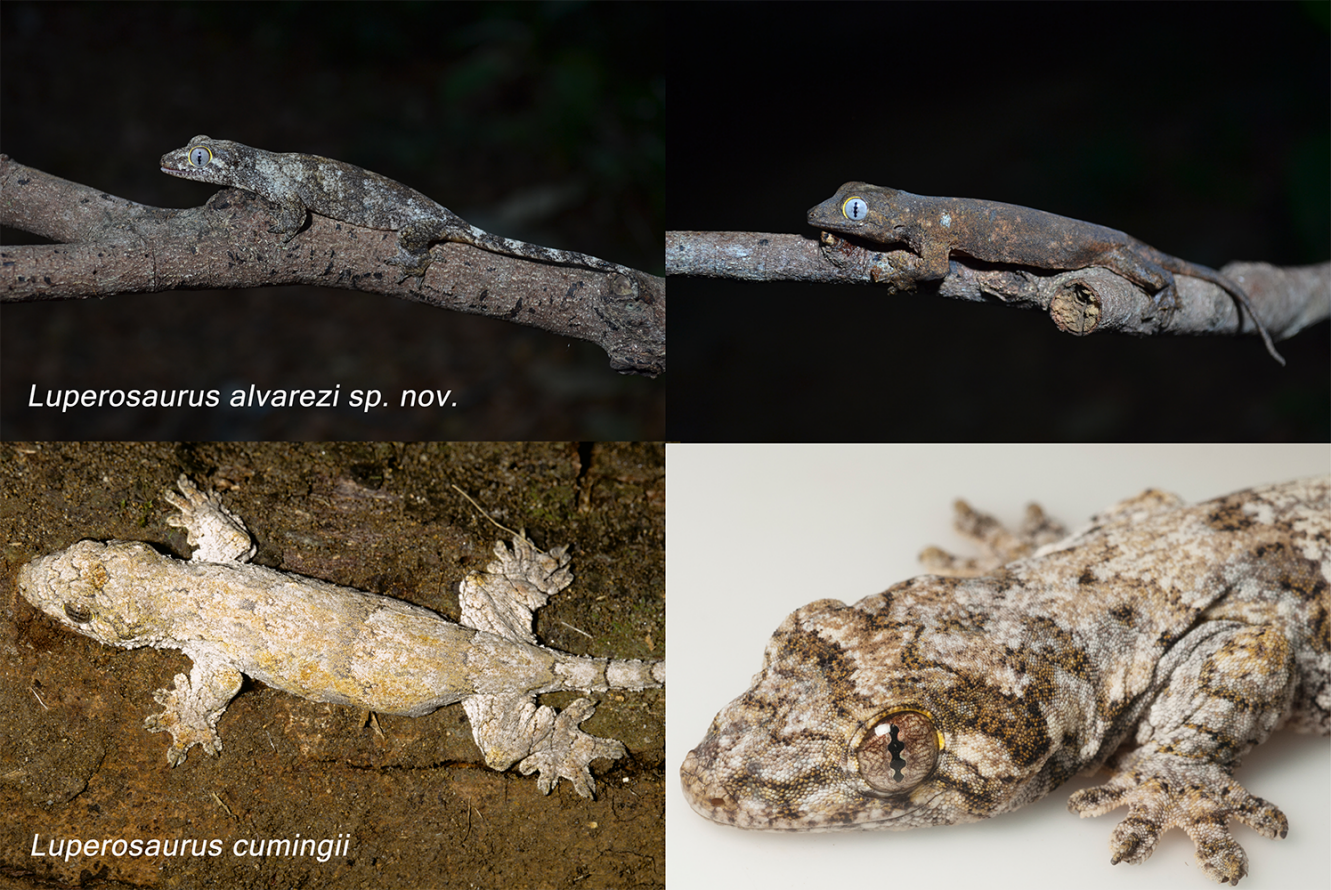
Photos in life of *Luperosaurus alvarezi.* sp. nov. (holotype and paratype) and *Luperosaurus cumingii*. Adult specimens of *Luperosaurus alvarezi* sp. nov. (left to right: holotype: PNM 9866: adult male; SVL 66.1 mm and paratype: UPLB-MNH-Z-NS 4622 (CGM 989); adult female; SVL 78.3 mm) from Mt. Guiting-Guiting Natural Park, Romblon Province, Sibuyan Island. Adult female *L* . *cumingii* (TNHC 61910; SVL 75.2 mm) from Mt. Malinao, Albay Province, Bicol Peninsula, Luzon Island. Note differences: *L*. *cumingii* has prominent spinose ventrolateral tail tubercles and heterogeneous nuchal and body tubercles (bottom left image). Photographs were taken by Camila G. Meneses and Rafe M. Brown.

**Figure 5:**
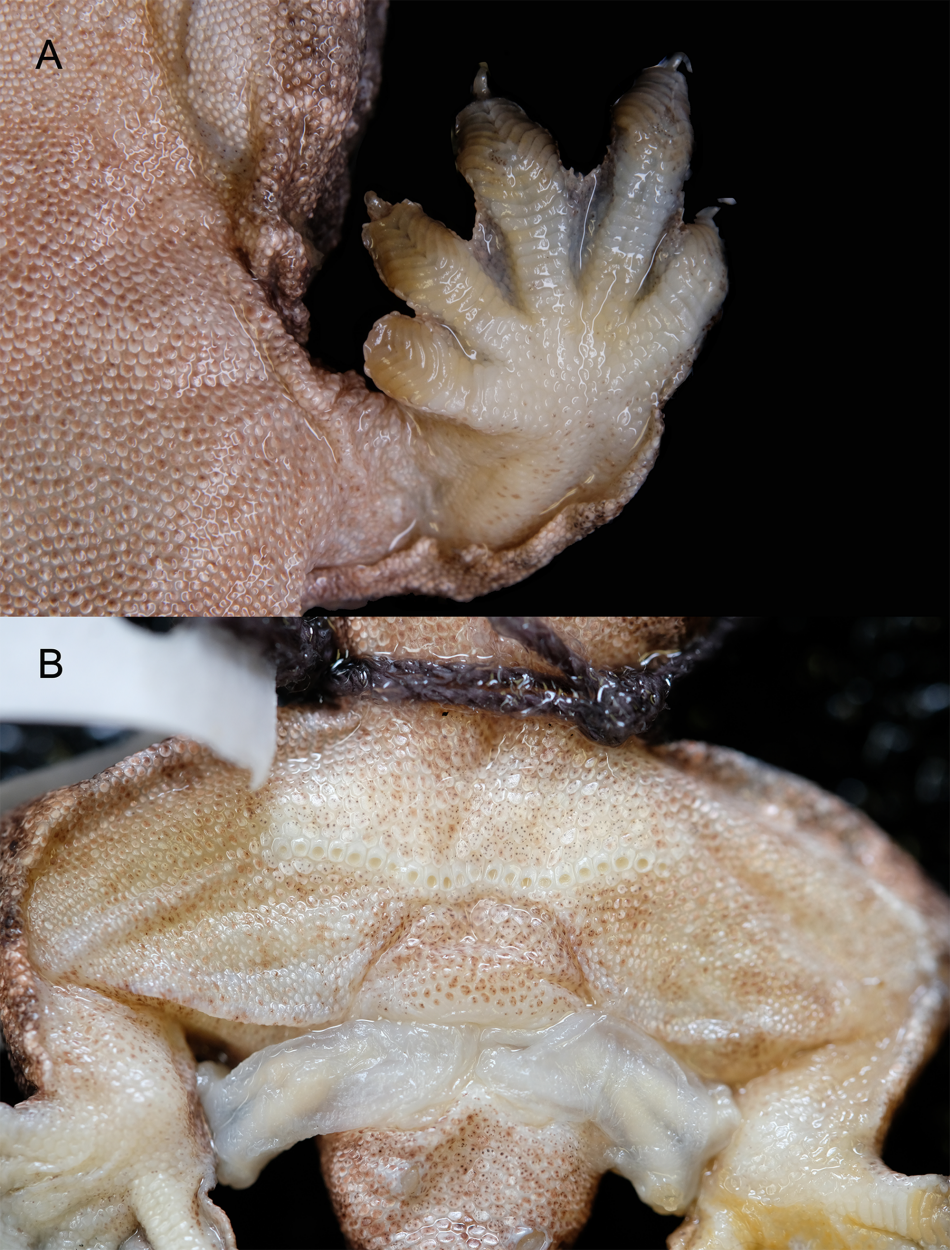
Ventral view of the forelimbs and male precloacal pores of *Luperosaurus alvarezi* sp. nov. Ventral aspect of (A) inferior view of left forelimbs of the adult male holotype of *Luperosaurus alvarezi* sp. nov. (PNM 9866) showing a well-developed interdigital webbing and moderate cutaneous expansions typical of anterior and posterior edges of forelimbs and (B) the holotype’s precloacal pores.

**Figure 6:**
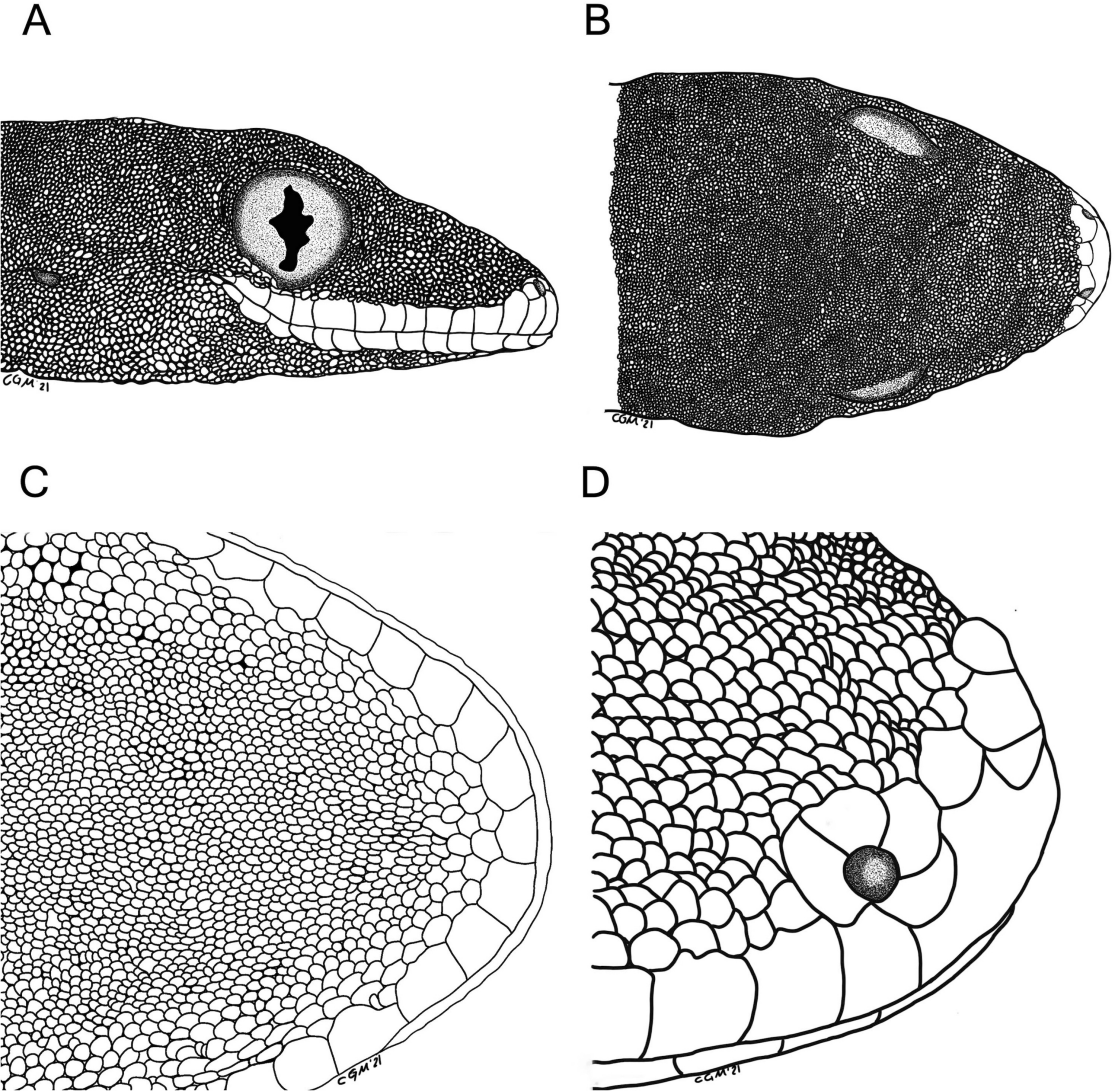
Illustrations of the head of *Luperosaurus alvarezi* sp.nov., showing dorsal, lateral, and ventral views. Illustration of the (A) Lateral (B) Dorsal (C) Ventral head view of the head, and (D) Snout view of the *Luperosaurus alvarezi* sp. nov. (PNM 9866).

**Figure 7:**
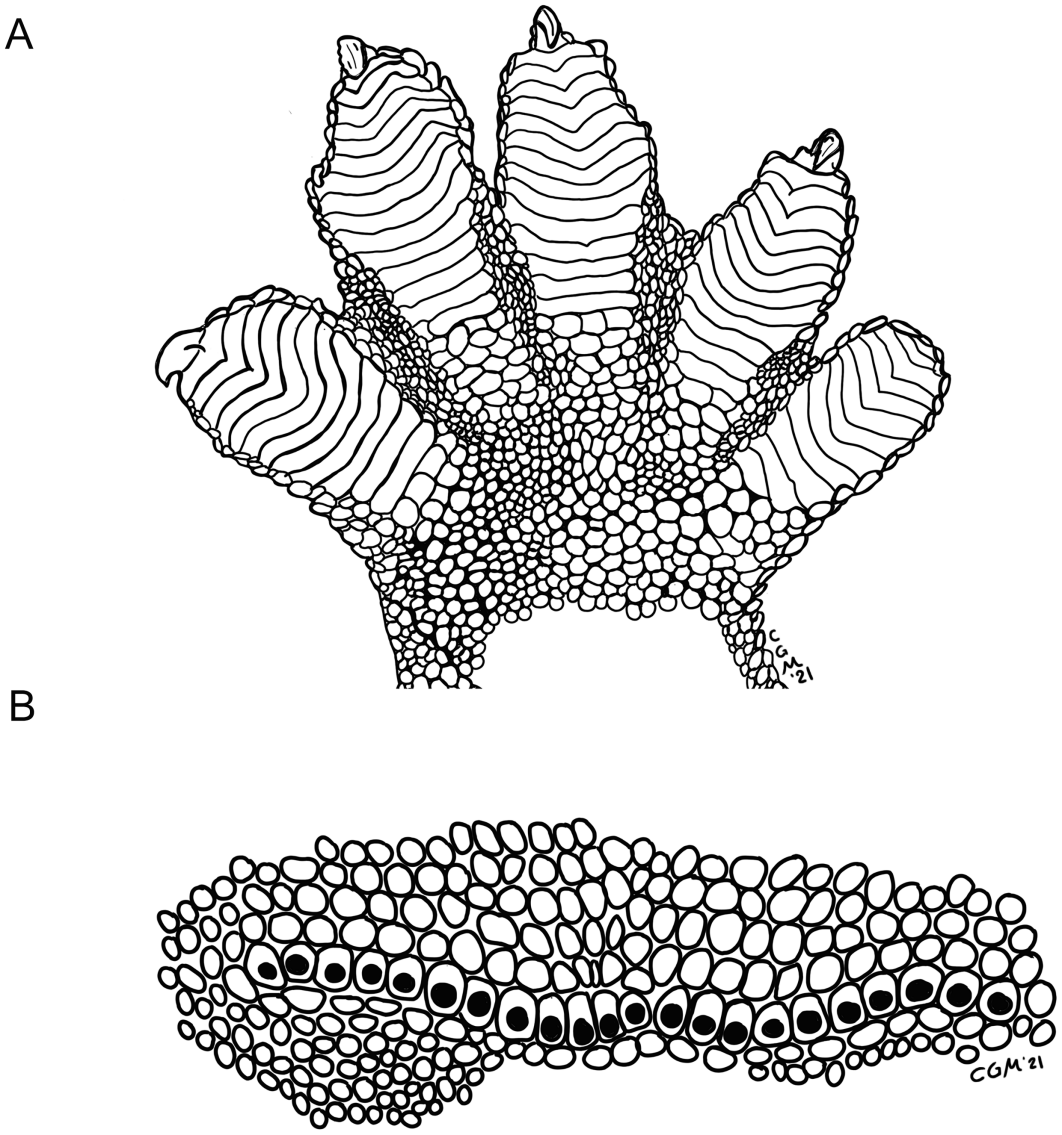
Holotype illustration of the manus and precloacal pores of *Luperosaurus alvarezi* sp. nov. Illustration of (A) palmar surface of right manus, and (B) precloacal pores of the *Luperosaurus alvarezi* sp. nov. (PNM 9866).

***Luperosaurus*** ***alvarezi*** **sp. nov.**
LSID: urn:lsid:zoobank.org:act:2C45E918-6610-4FCB-B8F5-71E1112D6DF3
Figures 3A, 3B, 4, 5A, 5B, 6A–6D, 7A, 7B

***Holotype***. PNM 9866 (Figs. 3A, 3B), formerly UPLB-MNH-Z-NS 4621 (CGM 988), small, but reproductively mature male (SVL 66.1 mm), collected 18 June 2017, between 0100 and 0200 hrs, from a vine overhanging the bank of the Gaong River, at Barangay Tampayan (12.4699°N, 122.5494°E), collected from 50 m a.s.l., Municipality of Magdiwang, Romblon Province, Sibuyan Island, Philippines, by Camila Meneses and Sean Arnel Gonzales.

***Paratype***. UPLB MNH-Z-NS 4622 (CGM 989), adult female (SVL 78.3 mm) bearing the same locality data as the holotype, collected between 2200 and 2400 hrs, from branches of a sapling tree overhanging the bank of Gaong River.

***Diagnosis.*** *Luperosaurus alvarezi* sp. nov. is diagnosed from congeners by possession of the following combination of characters: (1) bright yellow superciliaries and circumorbitals (Fig. 4); (2) light gray iris (Fig. 4); (3) precloacofemorals 22,22 (Fig. 5B; Fig. S1); (4) five scales contacting nostrils (Figs. 6A, 6B, 6D); (5) Head length/width 1.4,1.5; (6) presence of few, enlarged, flat to convex ornamental scales on margin of anterior forelimb expansion; (7) presence of elliptical, small, and oblique auricular opening; (8) dorsal body tubercles absent; (9) ventrolateral body tubercles absent; (10) presence of few, flat, and enlarged scales on caudal edges of tail whorls; (11) longitudinal midventrals 108, 110; (12) anterior hindlimbs expansions reduced to moderate folds. The condition of five scales contacting the nostrils, head length-to-width ratio, absence of dorsal and ventrolateral body tubercles, and presence of a few flat, enlarged scales on the caudal edges of tail whorls are shared among congeners but are diagnostic for *Luperosaurus alvarezi* sp. nov. in combination with other characters, distinguishing it from the morphologically similar *L. corfieldi* and *L. cumingii*, with which it shares certain intermediate external similarities. Table 1 presents a summary of the distribution of diagnostic character states among Philippine *Luperosaurus*.

***Comparisons.*** The critical comparison for the recognition of the new species is with *Luperosaurus corfieldi* (from the West Visayan islands of Panay and Negros), the taxon to which the *Luperosaurus alvarezi* sp. nov. specimens are most phenotypically similar and geographically proximate, as well as with *L. cumingii* (and to a lesser extent *L. angliit* and *L. macgregori*), a morphologically similar species distributed on adjacent landmasses of Luzon and its land-bridge satellite islands. Accordingly, we focus our comparisons on *L. corfieldi* and *L. cumingii* for the majority of our diagnosis. *Luperosaurus alvarezi* sp. nov. differs from *L*. *corfieldi* in having a higher number of precloacofemoral pores (*n* = 22 *vs.* 11–19), more scales contacting the nostrils (5 *vs.* 4), bright yellow superciliaries and circumorbital ring (*vs.* tan to pale yellow), and a light gray iris (*vs.* iris dark, tan to reddish brown; see Fig. 4). The new species exhibits a greater head length-to-width ratio (1.4–1.5 *vs.* 1.2), and is distinguished by the presence of a few enlarged, flat to convex ornamental scales on the margin of the anterior forelimb expansion (*vs.* absence), and an elliptical, small, obliquely oriented auricular opening (*vs.* large, subcircular). The new species further differs from *L. corfieldi* by the absence of dorsal and ventrolateral body tubercles (*vs.* presence of a few enlarged, convex tubercles), and the presence of ventrolateral tail tubercles consisting of a few enlarged, flat scales on the caudal edge of each tail whorl (*vs.* absent). Additional differences include fewer paravertebrals (179,182 *vs.* 218), ventral scales slightly enlarged, flat to granular, and subimbricate (*vs.* small, granular, and juxtaposed), slightly enlarged anterior-most chin scales (*vs.* small), fewer longitudinal midventrals (108–110 *vs.* 126), and a reduced anterior hindlimb cutaneous expansion (moderate flaps *vs.* wide flaps).

The new species differs from *L*. *cumingii* in having bright yellow superciliaries and a circumorbital ring (*vs.* yellow and gray), a light gray iris (*vs.* tan-gray with maroon reticulum), a greater head length-to-width ratio (1.4–1.5 *vs.* 1.2–1.3), and the absence of ornamental postrictal and nuchal tubercles (*vs.* numerous, highly spinose; Brown et al., 2011). It also differs in having an elliptical, small, obliquely oriented auricular opening (*vs.* oval, moderate, oblique), absence of dorsal body tubercles (*vs.* many, strong, and spinose), absence of ventrolateral body tubercles (*vs.* few, convex to spinose; Fig. 4), and presence of a few enlarged, flat scales on caudal edges of tail whorls (*vs.* absence). Additionally, *Luperosaurus alvarezi* sp. nov. has ventral scales that are slightly enlarged, flat to granular, and subimbricate (*vs.* small, granular, and juxtaposed), fewer paravertebrals (179,182 *vs.* 180–267), fewer longitudinal midventrals (108–110 *vs.* 124–165), and reduced anterior hindlimb expansions (moderate flaps *vs.* narrow flaps).

*Luperosaurus alvarezi* sp. nov. exhibits a reduced snout–vent length (SVL: 66.1, 78.3 mm) compared to *L. kubli* (105.4 mm), but its body size is apparently is larger than *L*. *angliit* (59.4–64.7 mm), *L*. *macgregori* (55.7–67.5 mm), *L*. *palawanensis* (43.7–52.0 mm), and *L. joloensis* (27.5–32.4 mm). The species has more precloacofemoral pores (22, 22) than *L. angliit* (17–19), *L*. *macgregori* (17–21), and *L*. *kubli* (16), but fewer than *L*. *palawanensis* (28–32) and *L*. *joloensis* (30–31). It also exhibits a higher number of scansors beneath Toe I (11, 12) and Toe IV (14, 16) than *L*. *angliit* (9–10; 11–13), *L*. *macgregori* (11–13; 13–16), *L*. *palawanensis* (9–11; 12–13), and *L*. *joloensis* (8–9; 9–13), with counts nearly identical to *L*. *kubli* (12; 16).

The presence of bright yellow superciliaries and circumorbital rings distinguishes *Luperosaurus alvarezi* sp. nov. from *L*. *angliit* (partly yellow and gray), *L*. *macgregori* (white), *L*. *palawanensis* (alternating brown and gold), *L. joloensis* (orange), and *L*. *kubli* (alternating brown and white). A light gray iris further distinguishes it from *L*. *angliit* (silver), *L*. *macgregori* (copper or orange), *L. palawanensis* (silver to gold), *L*. *joloensis* (silver), and *L*. *kubli* (silver). The presence of a single internasal contacting the rostral separates *Luperosaurus alvarezi* sp. nov. from most specimens of *L*. *angliit*, *L*. *macgregori*, and *L*. *palawanensis* (1–3 internasals), but not *L*. *joloensis* and *L*. *kubli* (1 internasal).

*Luperosaurus alvarezi* sp. nov. can be further distinguished by its head length-to-width ratio (1.4–1.5), which is greater than in *L*. *palawanensis* and *L*. *kubli* (1.2), but comparableto *L*. *angliit* (1.4–1.5), *L*. *macgregori* (1.4), and *L*. *joloensis* (1.3–1.4). Tail height-to-width ratio in *Luperosaurus alvarezi* sp. nov. (0.8) is greater than that observed in *L*. *joloensis* (0.5) but falls within the range of *L*. *angliit*, *L*. *palawanensis*, and *L. kubli* (0.7–1.0).

The presence of a few enlarged, flat to convex ornamental scales on the margin of the anterior forelimb distinguishes *Luperosaurus alvarezi* sp. nov. from *L*. *angliit*, *L*. *macgregori*, *L*. *palawanensis*, and *L*. *kubli* (all lacking this feature), and from *L*. *joloensis*, in which the trait is variably present (spinose) or absent. An elliptical, small, obliquely oriented auricular opening further separates *Luperosaurus alvarezi* sp. nov. from *L*. *angliit* (large, circular), *L*. *palawanensis* (large, subcircular), and *L*. *macgregori*, *L*. *joloensis*, and *L*. *kubli* (oval, small, oblique). The absence of dorsal body tubercles distinguishes *Luperosaurus alvarezi* sp. nov. from *L*. *palawanensis* (few, spinose, recurved) and *L*. *joloensis* (few, strongly spinose, recurved), while the lack of ventrolateral tubercles separates it from *L*. *macgregori* (few, convex), *L. palawanensis* (few, spinose), and *L*. *joloensis* (many, spinose). Caudal ornamentation in *Luperosaurus alvarezi* sp. nov. (few, flat, enlarged scales on the caudal edge of tail whorls) further distinguishes it from *L*. *macgregori* (absent or few enlarged scales), *L*. *palawanensis* (numerous, highly spinose, encircling each whorl), and *L*. *joloensis* (few, highly spinose, on the caudal edge of whorls).

The species has a greater number of midbody scales (152, 154) than *L. macgregori* (100–146), *L. palawanensis* (99–106), and *L. joloensis* (128–133), but fewer than *L. angliit* (162–173) and *L. kubli* (157). Slightly enlarged anterior-most chin scales separate *L. alvarezi* sp. nov. from *L. angliit* and *L. macgregori* (both with small undifferentiated chin scales). The wide anterior forelimb expansion in *L. alvarezi* distinguishes it from *L. angliit* (moderate flaps), *L. macgregori* (narrow flaps), *L. palawanensis* and *L. kubli* (absent), and *L. joloensis* (moderate flaps). Likewise, the wide posterior forelimb expansion contrasts with *L. angliit* (narrow flaps), *L. macgregori* (moderate), *L. palawanensis* (absent), *L. joloensis* (moderate), and *L. kubli* (minute flaps). Reduction of anterior hindlimb expansion (moderate flaps) contrasts with its absence in *L*. *macgregori*, *L*. *palawanensis*, and *L*. *kubli*. Finally, the wide posterior hindlimb expansion in *Luperosaurus alvarezi* sp. nov. contrasts with the moderate expansions in *L*. *angliit*, *L*. *macgregori*, and *L*. *kubli*, while *L*. *palawanensis* and *L*. *joloensis* also exhibit wide posterior expansions (see Table 1).

### Description of holotype

Adult male in excellent condition, undergoing ecdysis, with shedding skin visible on the dorsal and ventral surfaces of the head, body, manus, and pes (Fig. 3); small incision in the sternal region (liver sample was extracted and preserved for genetic material). Body relatively large, robust, 66.1 mm snout–vent, dorsally subcylindrical; head at widest point 0.2 times wider than body; trunk stout, venter flat; tail original, intact, relatively short, not depressed (subcylindrical in cross section); all limbs with expanded lateral cutaneous flaps or dermal folds covered with undifferentiated, flat, minute scales on dorsal and ventral surfaces; anterior margins of the forelimbs with wide flaps; posterior margins of forelimbs with wide flaps; anterior margins of hindlimb with moderate flaps; posterior margins of hindlimb with wide flaps.

Head moderate, large, stout; snout subelliptical, rounded at tip in dorsal and lateral aspect (Fig. 6); head width 70% of head length and 20% of snout–vent length; snout length 70% of head width and 40% of head length; dorsal surface of the head smooth, homogenous, with slight postnasal, prefrontal, interorbital, and parietal concavities; auricular opening elliptical, small, oblique; eye moderate; pupil vertical (Fig. 4; upper photos); tympanic annulus diameter 40% of eye diameter; limbs well developed, stout, robust, relatively muscular; femoral segments of hindlimb robust; tibia length 10% of snout–vent length, 70% of femur length.

Supralabials 14/14, bordered dorsally by one row of very slightly elongate, similarly flattened snout scales; infralabials 13/13; ventral chin scales only slightly enlarged; subsequent rows indistinguishable from uniformly small and granular gulars; postrictal and nuchal tubercles absent; mental small compared to adjacent infralabials.

Dorsal cephalic scales round, uniformly small, fine, granular, juxtaposed, and homogenous, except slightly enlarged scales on the distal portions of the snout, loreal, and prefrontal regions; preorbital region with slightly differentiated scales; temporal and parietal regions free of enlarged tubercles.

Axilla-groin distance 50% of snout–vent length; undifferentiated dorsal body scales, round, convex, nonimbricate, transversely undifferentiated, juxtaposed, lacking interstitial granules; transverse midbody scales 154 (including 33 slightly enlarged transverse ventrals; slightly enlarged, flat to granular, ventrals subimbricate; paravertebrals between limb insertion midpoints 179; longitudinal midventrals 108; dorsal body tuberculation absent; ventrolateral body tuberculation absent.

Dorsal scales of limbs, uniformly granular, small, round, flat to convex, juxtaposed, similar in size to dorsal trunk scales; forelimb scalation homogenous, tuberculation completely absent; scales of ventral surfaces homogenous, round, granular, juxtaposed, transitioning to scales extending onto the palmar surfaces of the manus and gradually increasing in size distal to digits; scales on ventral thigh surface similar, slightly enlarged near to groin; scales of ventral surface tibia and ankle moderately enlarged, flat, subimbricate, gradually transitioning to moderately enlarged scales on ventral side of pes; dorsal scales of the manus and pes similar to those scales on dorsal limb and trunk; dorsal and ventral scales on the cutaneous expansions bordering limbs minute, uniformly granular.

Enlarged, differentiated, and prominently dimpled scales of the precloacofemoral pore-bearing series (pores present in females but slightly dimpled; see Figs. 5B, 7B for male; Fig. S1 for both male and female) arranged in a continuous, nearly straight configuration across the precloacal region, and slightly extending laterally onto the distal portion of thigh; scales anterior to the precloacofemorals composed of one row of similarly sized, prominently dimpled scales, and two or three preceding rows that gradually decrease in size until becoming indistinguishable from surrounding ventrals in cloacal region (Figs. 5B, 7B; Fig. S1B); scales posterior to precloacofemorals undifferentiated.

Digits widely dilated; penultimate scansors deeply notched, not divided (Figs. 5A, 7A); extent of web between Toe III and IV 1/2; scales on dorsal surfaces of digits uniformly small, granular, juxtaposed; scales on ventral surfaces of digits transversely widened and slightly imbricate below proximal portions of digits; scales on ventral surfaces of interdigital webbing of both manus and pes uniformly small, granular, juxtaposed; Toe I scansors 11/11, Toe IV scansors 16/16; all digits except the first (inner) clawed; inner digits of both manus and pes with enlarged flattened nail in claw position; remaining terminal claw-bearing phalanges compressed, with large recurved claws rising free at distal end and extending onto the distal portion of the digit.

Tail relatively short, 70% of snout–vent length; tail height 80% of tail width; tail not depressed, subcylindrical, bearing flattened surface due to protrusion of ventrolateral edge composed of a few enlarged scales per tail segment (whorls); dorsal caudals, 191, uniformly granular; few, flat, scales enlarged, on caudal edge of each whorl; subcaudals 146/146, larger than dorsal caudal scales, subimbricate, flat to convex.

### Coloration of holotype in life

Dorsal surface of the head, extremities, midbody and tail of the holotype, was mottled dark brown. It has a rough, sparse, and uneven dark brownish color pattern (Fig. 4; see upper right photo). Superciliaries were bright yellow in life and exhibited bright yellow ventral coloration, with irregular, medially interrupted grayish-brown ventrolateral bands. Ventral surfaces of the head were yellow, with an irregular, medially interrupted grayish-brown ventrolateral bands. Iris coloration of a homogeneous light gray to blue, with no reticulate network (Fig. 4).

### Coloration of holotype in preservative

Dorsum medium brown, with white to pale brown blotches, a few blotched and indistinct transverse medium brown bands from mid-body to tail region, no irregular dark line patterns originating in different regions of the specimen’s body compared to other species were observed, dorsal tail coloration banding faintly with alternating light and dark brown (Figs. 3A, 3B).

Dorsal surfaces of limbs medium brown with no irregular lines or dark blotches on elbows or knees, limbs lacking transverse banding; dorsal surfaces of manus and pes medium brown, digits medium to light brown with dark brown distal portions; innermost digits slightly lighter; distal portions of tail not conspicuously lighter; ventral surface of tail medium brown with faint midventral stripe or transverse bands, same as the paratype (Fig. S2).

Infralabial region and chin flat cream to medium brown, throat region cream to light brown, flecked with yellow, dark brown ventrolateral jaw coloration; sternal region and venter medium brown to cream; ventral body light brown, a denser congregation of thin radiating dark brown lines on the ventrolateral body region—from its ventrolateral head down to its neck and mid-body region; ventral surfaces of limbs slightly lighter, nearly dirty cream; ventral surfaces of scansors of fingers and toes dark to pale yellow distal to proximal, respectively.

### Variation

The adult female paratype is very close in body size (SVL: 78.3 mm *vs.* 66.1 mm) and does not differ noticeably in body proportions from the male holotype. Based on the observed and measured morphological data, the female is bigger than the male (holotype: PNM 9866); however, our sampling is limited, and additional specimens are needed to determine if males are consistently smaller than females.

The color pattern of the adult female paratype (UPLB MNH-Z-NS 4622) is lighter than that of the holotype (Fig. 4, upper left photo). In life, the paratype had a gray dorsum with a dark chevron-like pattern traversing the body from scapular region to tail; dark brown to gray blotches were present on elbow and knee, and dorsal and lateral head surfaces with irregular dark streak from snout to nuchal region. Depending on color of surface on which live specimens originally rested (when first observed by CGM), middorsal surfaces were marked by thick dark brown transverse bands, giving individuals a very dark appearance, or by thick dark gray to brown bands with lighter gray interspaces forming more prominent bands and imparting a much lighter overall appearance. Distinct light and dark transverse bands were present on the dorsum and the tail was dark grayish-brown with five or six gray rings.

The uneven brown color pattern of the male holotype does not clearly differentiate it from the actual color of the female paratype, as the holotype was captured, photographed, and preserved when mottling (Fig. 4; see upper right photo). Hence, definitive color variation for this species warrants additional study. The observed pattern in the paratype suggests that the holotype might also have a grayish- to greenish-brown big chevron pattern extending along the body and onto the tail.

In life, both specimens, had bright yellow superciliaries and circumorbitals and exhibited bright yellow ventral coloration with irregular, medially interrupted grayish-brown ventrolateral bands. Ventral surfaces of the head were yellow, with similar irregular, medially interrupted grayish-brown ventrolateral bands. Both specimens had irises of a homogeneous light gray to blue without reticulate network (Fig. 4).

### Distribution and Natural History

*Luperosaurus alvarezi* sp. nov. is known only from the type locality, a protected forested area surrounding Mt. Guiting-Guiting National Park, in central Sibuyan Island, Romblon Island Group (Romblon Province; Fig. 1). Based on geological history and channel depth (Siler et al., 2016; Voris, 2000; Yumul Jr et al., 2003; Yumul et al., 2009), it remains an open question, whether this species could be (or may have once been) distributed in appropriate habitats of Romblon and Tablas islands, the small islands to the west of Sibuyan, which were connected to one another, but not to the deep water island of Sibuyan. We collected both specimens on the same night, in near identical microhabitats (river-side tree trunks). However, they were conspicuously absent over weeks of nocturnal surveys, in the same and adjacent habitats, extending before and after the date of collection. Multiple earlier surveys likewise failed to find *Luperosaurus* on Sibuyan (Brown & Alcala, 1974; Siler et al., 2012; Meneses et al., 2022). However, whereas no hint of *Luperosaurus* has ever been reported previously from Sibuyan or other islands of Romblon Province (Romblon, Tablas). *Luperosaurus corfieldi* (Gaulke, Rösler & Brown, 2007; Gaulke, 2011) is known from northwest Panay, immediately to the south of the Romblon Island Group (see map, Fig. 1).

Sympatric gekkonid species of Sibuyan Island include *Cyrtodactylus philippinicus, Gekko gecko*, *G*. *coi, G*. *mindorensis, Gehyra mutilata, Hemidactylus frenatus, H*. *platyurus, Lepidodactylus* sp.*, L*. *lugubris,* and *Pseudogekko isapa* (Brown, Diesmos & Oliveros, 2011; Brown et al., 2011; Siler et al., 2012; Siler et al., 2016; Meneses et al., 2020; Meneses et al., 2022). If the new species also occurs on Tablas and/or Romblon islands, it may also be sympatric with *Gekko romblon*.

### Etymology

We are pleased to name the new species after our dear friend, frequent field companion and collaborator, the late James Alvarez, who lost his life while conducting bat research in the Philippines’ highest mountain, Mt. Apo, on December 8, 2018. We derived the specific epithet, a patronym, in the genitive singular, in recognition to Mr. Alvarez’s scientific contributions and demonstrated personal commitment to furthering knowledge of the natural history of Philippine chiropterans—in particular, the ecology and diversity of bats in Sibuyan Island.

### Phylogenetic analyses

**Figure 8:**
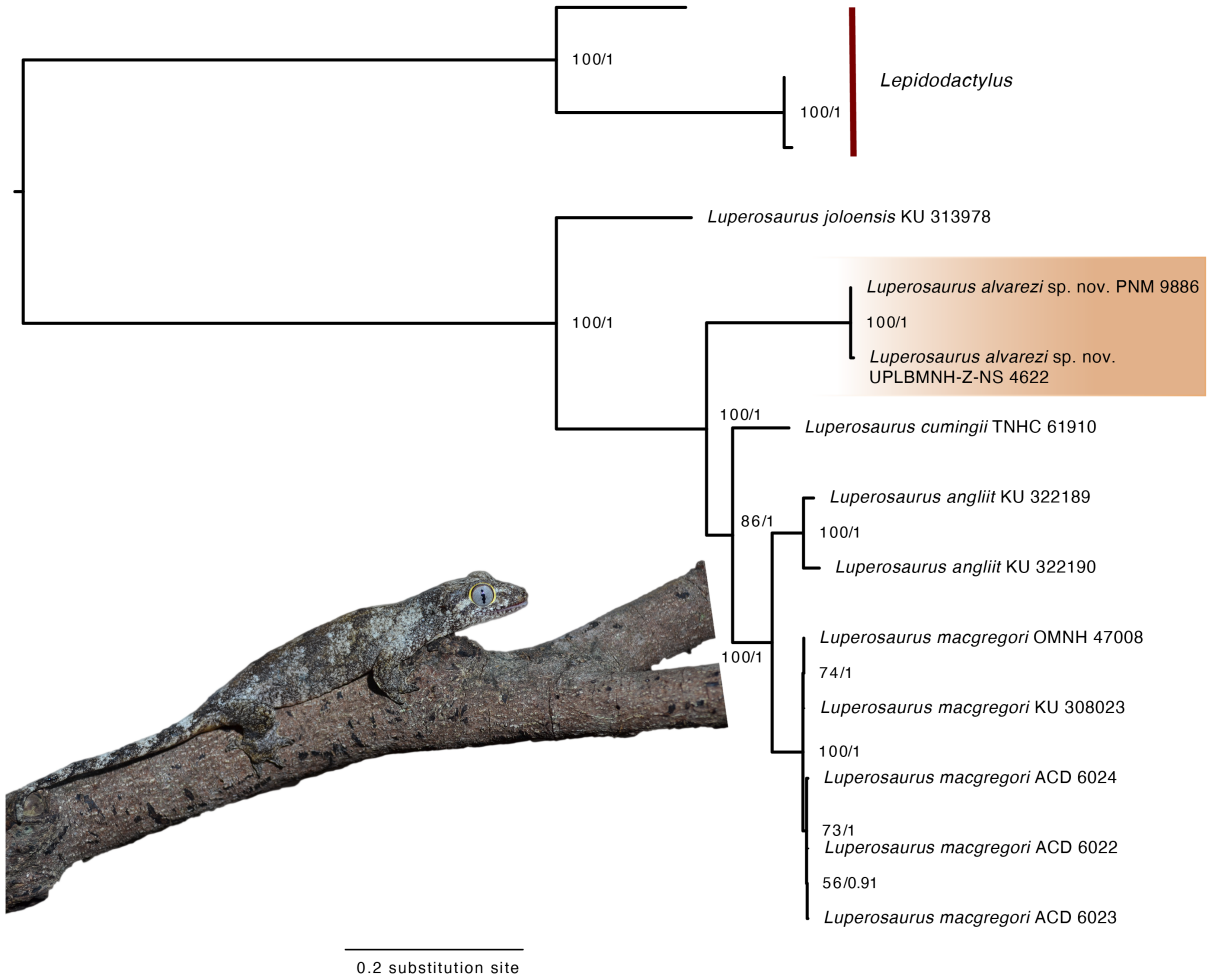
Maximum clade credibility tree for *Luperosaurus alvarezi* sp.nov. inferred from IQ-TREE and BEAST analyses, showing bootstrap values and posterior probabilities at nodes. Inferred maximum likelihood tree estimated using IQ-TREE and BEAST v2.7.6 derived from ND2 data for * Luperosaurus alvarezi* sp. nov. Numbers on the nodes denotes ultrafast bootstrap (BS) support values (BS: high branch support = 95–100%; moderate to low branch support 70–94%) and posterior probability (PP) support values (PP: high branch support = 0.95; moderate to low branch support 0.70 –0.94). Position of *Luperosaurus alvarezi* sp. nov in orange. Photograph by Camila G. Meneses.

Although we do not use genetic distance estimates to justify recognition of the new species described here, analyses of mitochondrial ND2 sequence data resulted in topologies consistent with the hypothesis that *Luperosaurus alvarezi* sp. nov. represents a distinct evolutionary lineage within the genus *Luperosaurus*. ML & BI approaches resulted in identical (congruent) topologies, with moderate to strong support for all nodes (Fig. 8; Fig. S3).

*Luperosaurus alvarezi* sp. nov. was recovered as the earliest-diverging lineage in the Maximum Likelihood tree forming a well-supported monophyletic clade represented by two specimens (PNM 9866 and UPLB-MNH-Z-NS 4622; UFBootstrap = 100%; posterior probability = 1.0). The placement of *Luperosaurus alvarezi* sp. nov. at the base of phylogenetic tree estimate suggests a uniquely early-diverging lineage within the Philippine members of the genus. Our maximum likelihood and Bayesian analyses both strongly support the monophyly of *Luperosaurus alvarezi* sp. nov., as well as its placement with the Philippine *Luperosaurus* clade, along with closely related species from Sibuyan‘s closest adjacent island, Luzon (Fig. 8; Fig. S3).

Both analyses unsurprisingly continue to provide robust support for the monophyly of genus *Luperosaurus*, and we interpret the substantial branch length separating *Luperosaurus alvarezi* sp. nov. as consistent with the hypothesis of its evolutionary distinctiveness from its congeners.

Our findings align with previous phylogenetic studies of Philippine gekkonids (*e.g.*, Siler et al., 2014; Wood Jr et al., 2020) and, with the caveat that *L. corfieldi* remains unsampled for molecular data, additionally suggest that *Luperosaurus alvarezi* sp. nov. appears most closely related to species found on Luzon and its neighboring land-bridge islands, such as *L*. *cumingii* (Southern Luzon), *L*. *angliit* (Eastern Luzon), and *L*. *macgregori* (Babuyan Islands, north of Luzon). Future studies should prioritize inclusion of sequence data from *L*. *corfieldi* for a more comprehensive phylogenetic estimate of species-level relationships within the Philippine clade.

## Discussion

It is not surprising that Sibuyan Island (and possibly other landmasses among the Romblon Island Group (RIG)) has harbored an undetected and undescribed *Luperosaurus.* First, the extreme biogeographical isolation of Romblon Island Group landmasses certainly would be expected to foster coalescence of independent and reproductively cohesive evolutionary lineages (Wiley, 1978; Frost & Hillis, 1990; De Queiroz, 1999; De Queiroz, 2005). Second, multiple other vertebrates (including gekkonids) are known to be endemic to the larger island group or Sibuyan itself (Brown, Diesmos & Oliveros, 2011; Brown et al., 2011; Siler et al., 2012; Siler et al., 2016; Meneses et al., 2020; Meneses et al., 2022). Accordingly, we recognize the Sibuyan population as a new species, given its unambiguously clear phenotypic distinctiveness and numerous fixed (discrete) character state differences which foster its diagnosis it (Table 1) from the most geographically proximate (*L*. *corfieldi*) and phenotypically most-similar (*L*. *angliit* and *L*. *cumingii*) Philippine *Luperosaurus*. The new species’ phylogenetic placement (the first-branching lineage among core Philippine clade *Luperosaurus* species) does not contradict our taxonomic findings.

The results of our phylogenetic analyses and consideration of external phenotypic characteristics unambiguously support our contention that the new species, *Luperosaurus alvarezi* sp. nov., is a member of the core Philippine clade of *Luperosaurus.* Other confirmed members of the “true” *Luperosaurus* include *L*. *angliit*, *L*. *corfieldi*, *L*. *macgregori*, and the type species for the genus, *L. cumingii* (Gray, 1845; Fig. 8). Although we expect the morphologically similar species *L*. *kubli* (known, to date from a single specimen and without corresponding genetic material; Brown, Diesmos & Duya, 2007) to eventually prove to be a member of this clade as well, uncertainty surrounds the phylogenetic placement of Philippine *L*. *palawanensis* (no genetic material available; Brown & Alcala, 1978; Jose et al., 2020), and *L*. *joloensis* (Taylor, 1918; Brown & Alcala, 1978; Brown & Diesmos, 2000), with the latter unrelated to true *Luperosaurus*, but nested in the broader clade reffered to *Lepidodactylus* (Oliver et al., 2018).

Based on surprising, unpredicted phylogenetic relationships, other former members of the genus *Luperosaurus* (*L*. *gulat, L*. *browni, L*. *brooksii,* and *L*. *iskandari*) have already been transferred to the genus *Gekko* (Russell, 1979; Ota, Sengoku & Hikida, 1996; Das, Lakim & Kandaung, 2008; Brown et al., 2012; Oliver et al., 2018; Wood Jr et al., 2020). *Luperosaurus* has been long recognized as a genus characterized by a confusing blend of pleisiomorphic and apomorphic phenotypic characters, otherwise apparently diagnostic of *Gekko, Lepidodactylus,* and *Pseudogekko* (Gray, 1845; Boulenger, 1885; Taylor, 1922; Brown, 1964; Kluge, 1968; Russell, 1979; Brown, Supriatna & Ota, 2000; Brown, Diesmos & Duya, 2007; Brown et al., 2010).

The foremost issue, with respect to the identification and diagnosis of the new species, was to distinguish it from the three most similar and geographically proximate forms: *L. corfieldi* (from Panay and Negros islands), *L. angliit*, and *L*. *cumingii* (both from Luzon Island). The Sibuyan form can readily be diagnosed from its closely related congeners, *L. angliit* and *L*. *cumingii*, of the Luzon PAIC, and *L*. *corfieldi*, of the West Visayan PAIC, based on non-overlapping ranges in continuous and meristic morphological characters, and discrete character state differences in traditional categorical characters (Table 1). For example, *Luperosaurus alvarezi* sp. nov. possesses a high number of precloacofemoral pores; a light gray iris; five scales contacting nostrils; an intermediate head length/width ratio; the presence of a small, obliquely elliptical auricular opening; dorsal tubercles absent; ventrolateral body tubercles absent; and narrow folds in the anterior hindlimbs expansion. *Luperosaurus alvarezi* sp. nov. appears phenotypically most similar to *L*. *corfieldi*, and, to a lesser extent, to *L*. *cumingii* (Table 1).

We suspect that additional species of core Philippine *Luperosaurus* remain to be discovered if small islands, isolated mountain ranges, and intact patches of coastal habitats can be sampled correctly in the future. Although this challenge may be hampered by widespread degradation of forested habitats, logistical obstacles to fieldwork, and increasing bureaucracy (Brown, Diesmos & Oliveros, 2011; Brown, Diesmos & Alcala, 2002; Brown, 2015; Gonzalez et al., 2018), we have nevertheless been pleasantly surprised to find *Luperosaurus* species persisting in small patches of original forest and/or second-growth vegetation adjacent to original forest (Brown, Diesmos & Oliveros, 2011; Oliver et al., 2020).

Upon originally describing *Luperosaurus corfieldi* from Panay Island, Gaulke, Rösler & Brown (2007) commented on previous (at that time) inaccuracies associated with recognizing *L*. *cumingii* as a “widely distributed” species. At the time, *L*. *cumingii* had been reported from isolated populations separated from two distinct PAICs (Brown, Diesmos & Alcala, 2002; Brown & Diesmos, 2009) Luzon and West Visayas, plus two deep-water small islands (Lubang Island, northwest of Mindoro Island; and Camiguin Sur Island, northeast of Mindanao Island), associated with two additional PAICs (Mindoro and Mindanao, respectively). Thus, *L*. *cumingii* truly seemed to be a widespread species (Gaulke, Rösler & Brown, 2007). However, given that so many comprehensive phylogenetic studies of archipelago-wide Philippine clades have found highly partitioned groups of endemic species, with geographic ranges corresponding to former Pleistocene sea shores (Brown & Guttman, 2002; Evans et al., 2003; Brown, Supriatna & Ota, 2000; Brown, McGuire & Diesmos, 2000; Brown et al., 2016; Brown et al., 2017), it seemed reasonable to expect a highly specialized forest-interior group of geckos (Oliver et al., 2018; Oliver et al., 2020) to conform to the biogeographic history of the archipelago, and to exhibit patterns reminiscent of many other co-distributed taxa (Brown & Diesmos, 2009; Brown et al., 2013). Thus, Gaulke, Rösler & Brown (2007) had little hesitation in referring the population from the Luzon satellite Polillo Island to *L*. *cumingii* (represented by a single hatchling; Taylor, 1922), and considering the Panay Island population a new species (*L*. *corfieldi*). Gaulke, Rösler & Brown (2007) also emphasized the suspected distinctiveness of the other populations known at the time but stated that they were represented by too few specimens to be assessed taxonomically. Later, with the description of *L*. *angliit* and the discovery of *Luperosaurus* on Camiguin Norte Island (north of Luzon), Brown, Diesmos & Oliveros (2011) reasoned that the older European specimens from “Camiguin” might likely have originated on Camiguin Norte Island, not Camiguin Sur—thus potentially eliminating the Mindanao PAIC from further consideration as part of the geographic distribution of *L. cumingii*.

In the intervening years, *L*. *corfieldi* has been rediscovered on Negros Island (confirming the West Visayan PAIC endemicity predicted by Gaulke, Rösler & Brown (2007)), the Camiguin Norte Island + northern Luzon population has been recognized as *L*. *angliit* (Brown, Diesmos & Oliveros, 2011), and true *L*. *cumingii* has been recorded on multiple occasions from southern Luzon (Laguna and Quezon provinces), and Luzon’s Bicol Peninsula (R. Brown, 2025, unpublished data; Oliver et al., 2020)—vindicating three of the four predictions asserted by Gaulke, Rösler & Brown (2007). There is still an unresolved question posed by Gaulke, Rösler & Brown (2007), which involves the status of the Lubang Island population. The population has been collected only once, and is represented in collections solely by a single, incomplete subadult male specimen (PNM 7242), which was nonetheless noted as potentially phenotypically distinct (Gaulke, Rösler & Brown, 2007; Dolino et al., 2009). Every effort should be made to resurvey Lubang Island and/or the adjacent coastal habitat of the southern Zambales Mountains of Luzon for *Luperosaurus*, which may represent another undescribed species.

The secretive behavior and rarity of the *Luperosaurus* lizards continue to present a challenge to field researchers (Oliver et al., 2020). For years, species of *Luperosaurus* were misunderstood, their numbers underestimated (Gaulke, Rösler & Brown, 2007; Brown & Diesmos, 2000; Brown, Diesmos & Duya, 2007; Brown, Diesmos & Oliveros, 2011), and our collective understanding of phylogenetic relationships radically shifted with each new analysis (Brown & Diesmos, 2000; Brown et al., 2012; Heinicke et al., 2012; Oliver et al., 2018). With the unexpected or accidental discovery of another new species, or a new type of data applied, the biology, natural history, relationships, and microhabitat preferences of *Luperosaurus* have become incrementally clearer (Brown & Diesmos, 2000; Brown, McGuire & Diesmos, 2000; Brown, Diesmos & Duya, 2007; Brown, Diesmos & Oliveros, 2011; Brown et al., 2012; Oliver et al., 2020; Wood Jr et al., 2020).

The discovery of *Luperosaurus alvarezi* sp. nov. on Sibuyan Island, particularly within the Mt. Guiting-Guiting Natural Park, offers an opportunity to study a species of *Luperosaurus* further, from within a known, protected, accessible area for which other associated biodiversity data are available (Heaney, 1985; Goodman, Willard & Gonzales, 1995; Heaney & Regalado, 1998; Tumaneng et al., 2015). Nonetheless, threats to the forested habitats of *L uperosaurus alvarezi* sp. nov. exist in the form of incessant anthropogenic disturbances such as tourism, logging, mining, and road development. The existence of such a rare, secretive forest-obligate species from within the borders of Mt. Guiting-Guiting Natural Park adds to the weight of evidence that should be utilized to convince policymakers, various stakeholders, local communities, and conservationists to work together and strengthen strategies for sustainable development and conservation of Sibuyan Island’s natural resources. This discovery further emphasizes the degree of faunal diversity and endemism of the Romblon Island Group. We urge immediate, comprehensive, and more extensive herpetological inventory efforts on all islands of the province, extending to nearby landmasses—particularly to any remaining unprotected forests on Tablas and Romblon islands, and also to any small islands adjacent to these larger landmasses. All such habitats could contribute to the last vestiges of habitat of *Luperosaurus alvarezi* sp. nov., or represent the only habitat left for other, undiscovered and unrecognized Philippine *Luperosaurus* species on the verge of extinction.

## Supplemental Information

10.7717/peerj.20504/supp-1 Supplemental Information 1 Precloacal and femoral pores of the female paratype (UPLBMNH-Z-NS 4622) and male holotype (PNM 9866) of *Luperosaurus alvarezi sp. nov.* Comparison of the precloacal–femoral region in female and male *Luperosaurus alvarezi* sp. nov. The female (UPLBMNH-Z-NS 4622, collected from Mt. Guiting-Guiting, Romblon Province, Sibuyan Island, Philippines) shows indistinct or poorly defined precloacal–femoral pores, whereas the male (bottom; PNM 9866, collected from thte same locality exhibits clearly developed pores in the same anatomical position near the cloacal–femoral region.

10.7717/peerj.20504/supp-2 Supplemental Information 2 Female paratype of *Luperosaurus alvarezi* sp. nov. in preservative Female specimen of *Luperosaurus alvarezi* sp. nov. (UPLBMNH-Z-NS 4622) collected from Mt. Guiting-Guiting Natural Park, shown in dorsal and ventral views to illustrate general morphology and preserved coloration.

10.7717/peerj.20504/supp-3 Supplemental Information 3 Bayesian inference tree of *Luperosaurus alvarezi* sp. nov. showing posterior probabilities at nodes Bayesian inference tree estimated from 14 ND2 mitochondrial gene samples of *Luperosaurus*. Bayesian posterior probabilities are shown at nodes, with 1.00 indicating 100% support. The scale bar represents the number of substitutions per site.

10.7717/peerj.20504/supp-4 Supplemental Information 4 Appendix, specimen summary table with GenBank accession numbers, and supplementary figures

10.7717/peerj.20504/supp-5 Supplemental Information 5 Aligned sequence dataset in FASTA format used for the analyses

10.7717/peerj.20504/supp-6 Supplemental Information 6 Text file of Luperosaurus alvarezi ND2 sequences submitted to GenBank with assigned GenBank accession number

## Additional Information and Declarations

### Field Study Permissions

The following information was supplied relating to field study approvals (i.e., approving body and any reference numbers): Fieldwork was conducted under a Memorandum of Understanding between the University of Kansas, the University of the Philippines Los Baños (UPLB), and the UPLB-Museum of Natural History; a Wildlife Gratuitous Permit to Collect (No. MIMAROPA-2017-0005); and Institutional Animal Care and Use Committee (IACUC) approval (158-04) from the University of Kansas, granted to CGM and RMB, respectively.

### Data Availability

The following information was supplied regarding data availability: The ND2 sequence data are available at GenBank: PX262121 to PX262129.

### New Species Registration

The following information was supplied regarding the registration of a newly described species: Publication LSID: urn:lsid:zoobank.org:pub:90E18669-18E2-4EB8-A10A-3FC1591F2B87 Luperosaurus alvarezi Species LSID: urn:lsid:zoobank.org:act:2C45E918-6610-4FCB-B8F5-71E1112D6DF3
