# Supplementary figures and images for: A new species of fringed Forest Gecko, genus Luperosaurus (Squamata: Gekkonidae), from Sibuyan Island, Central Philippines
Source: PeerJ. 2026 Mar 4;14:e20504. doi: 10.7717/peerj.20504 (PMC12967072; doi:10.7717/peerj.20504)

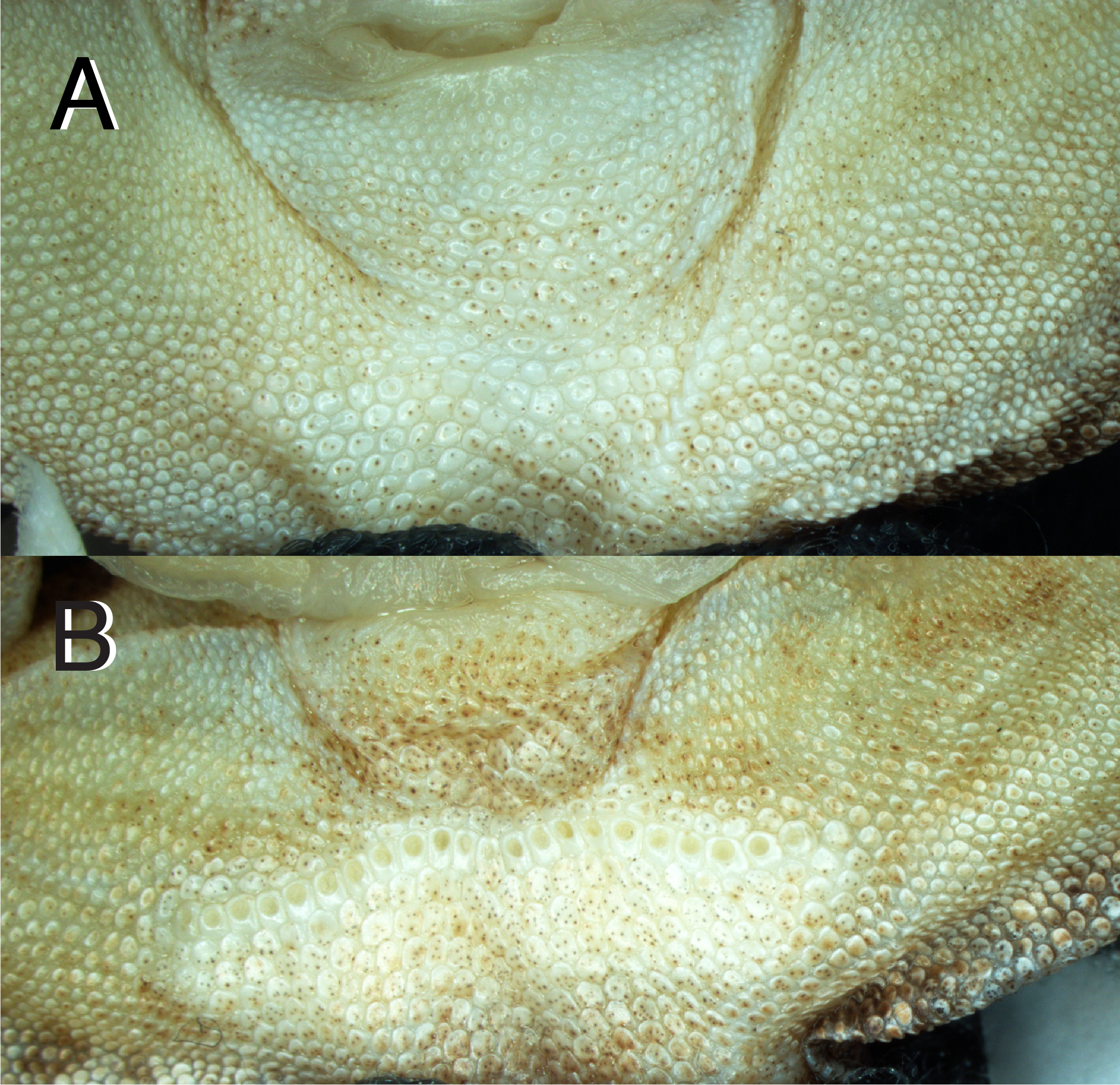

Supplement: Supplemental Information 1 — Comparison of the precloacal–femoral region in female and male Luperosaurus alvarezi sp. nov. The female (UPLBMNH-Z-NS 4622, collected from Mt. Guiting-Guiting, Romblon Province, Sibuyan Island, Philippines) shows indistinct or poorly defined precloacal–femoral pores, whereas the male (bottom; PNM 9866, collected from thte same locality exhibits clearly developed pores in the same anatomical position near the cloacal–femoral region. [file peerj-14-20504-s001.png]

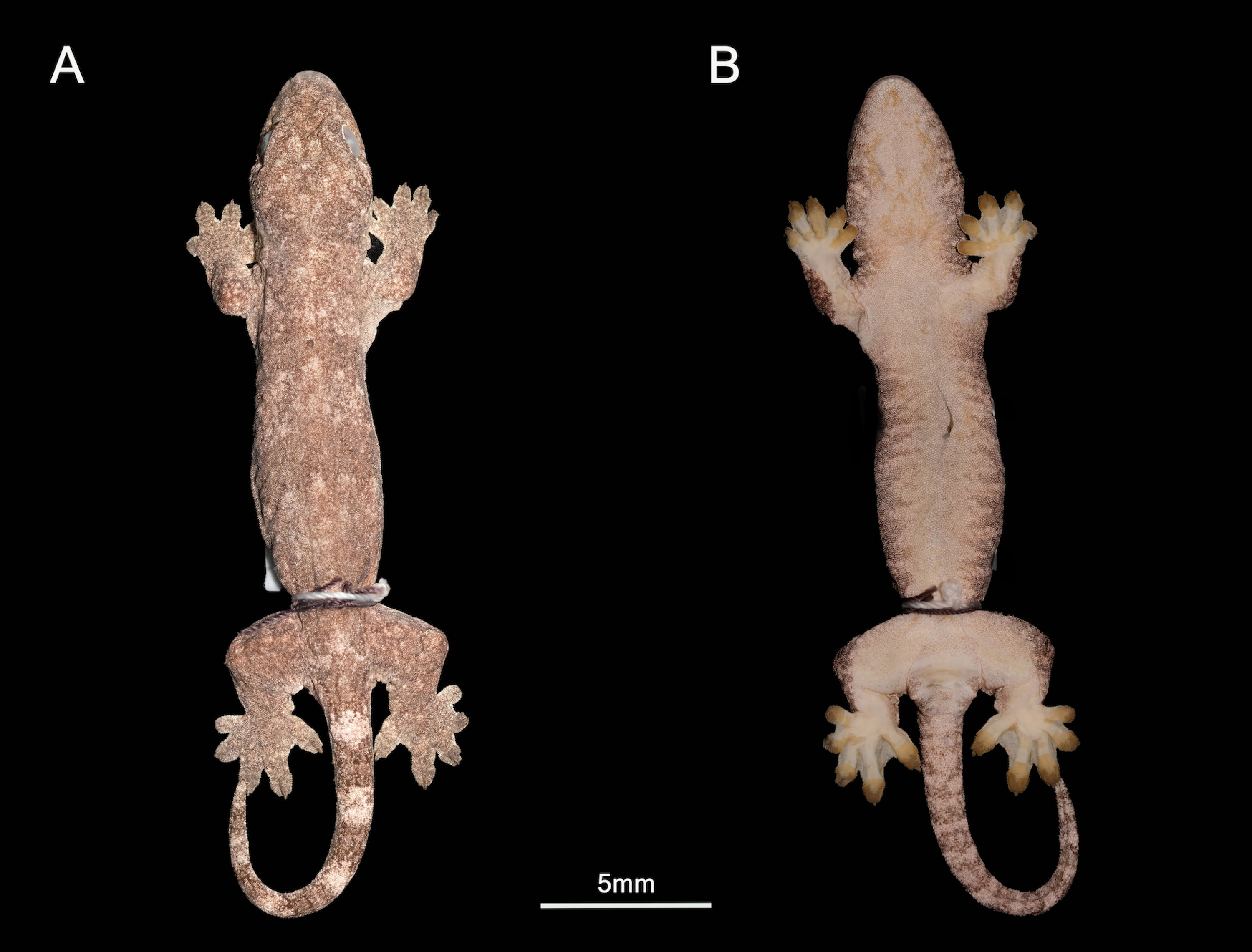

Supplement: Supplemental Information 2 — Female specimen of Luperosaurus alvarezi sp. nov. (UPLBMNH-Z-NS 4622) collected from Mt. Guiting-Guiting Natural Park, shown in dorsal and ventral views to illustrate general morphology and preserved coloration. [file peerj-14-20504-s002.png]

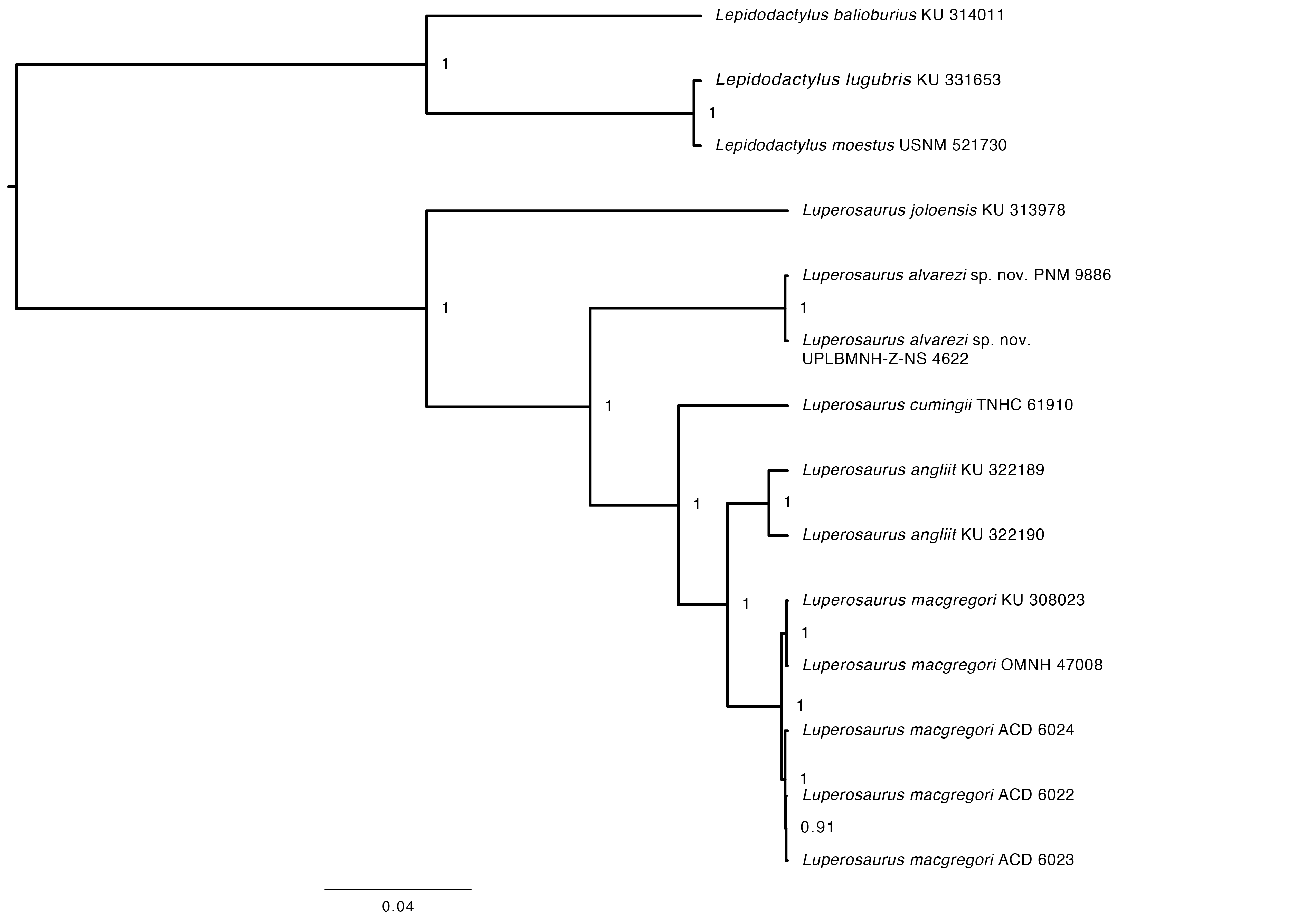

Supplement: Supplemental Information 3 — Bayesian inference tree estimated from 14 ND2 mitochondrial gene samples of Luperosaurus. Bayesian posterior probabilities are shown at nodes, with 1.00 indicating 100% support. The scale bar represents the number of substitutions per site. [file peerj-14-20504-s003.png]
