# Supplementary material for: A new species of fringed Forest Gecko, genus Luperosaurus (Squamata: Gekkonidae), from Sibuyan Island, Central Philippines
Source: PeerJ. 2026 Mar 4;14:e20504. doi: 10.7717/peerj.20504 (PMC12967072; doi:10.7717/peerj.20504)

**Appendices**

**Appendix 1: Specimen Examined**

*Luperosaurus angliit*: Philippines, Luzon Island, Aurora Province, Municipality of

Baler, Barangay Zabali, Aurora State College of Technology campus: PNM 9702 (holotype); Laguna Province, Municipality of Los Baños, Barangay Bating Malaki, UPLB Forestry Campus; KU 326160; Municipality of Casiguran, Barangay Culat, Sitio Casapsapan: KU 321815, PNM 9703 (paratype).

*Luperosaurus* *corfieldii*: Philippines, Panay Island, Aklan Province, Municipality of Buruanga, Barangay Tagosip: PNM 7919 (holotype), 7920, 8489 (paratypes); Negros Isl., Negros Oriental Prov., Municipality of Valencia, Lake Balinsasayao: SUR 2211; Saksak Cr., Mt. Cuernos de Negros, "Camp Lookout:" CAS-SU 24394–95; Negros Isl., Lake Balinsasayao: CAS 182570.

*Luperosaurus cumingii*: Philippines, Luzon Island, Camarines Sur Province, Municipality of Caramoan, Anuling Mountain: UF 77829; Albay Province, Municipality of Tiwi, Barangay Banhaw, Sitio Purok 7, Mt. Malinao: TNHC 61910; Luzon Island, Quezon Province, Municipality of Infanta, Barangay Magsaysay, Infanta-Marikina Highway, Southern Sierra Madre Mountain Range: KU 275883, 275884; RMB 26068, 26069, 26094, 26108, 26134, and 26135; Quezon Province, Municipality of Siniloan, Barangay Magsaysay, University of the Philippines Quezon Land Grant, Lalawinan River Drainage: KU 345671; "Central Luzon, Philippines:” SMF 9044.

*Luperosaurus* cf. *cumingii*: Lubang Island, Mindoro Oriental Province, Municipality of Lubang: PNM 7242.

*Luperosaurus* *joloensis*: Philippines, Mindanao Isl., Cotobato Province; "Cotobato Coast:" MCZ 26118; Zamboanga City Province, Municipality of Zamboanga City; Barangay Pasanonca, Sitio Canucutan, Pasanonca Natural Park: KU 314947; Sulu Archipelago, Jolo Isl., Siet Lake: CAS 60675 (paratype).

*Luperosaurus kubli*: Philippines, Luzon Island, Quirino Province, Muncipality of Nagtipunan, Barangay Disimungal, Mt. Lataan (Sierra Madre Mountain Range): PNM 9156.

*Luperosaurus* *macgregori*: Philippines; Batan Island, Batanes Province, Municipality of Basco, Barangay San Antonio, Sitio Chadpidan; KU 314021; Calayan Island: CAS-SU 6263 (paratype); Cagayan Province, Babuyan Island Group., Barit Island (near Fuga Island): USNM 508306—508308; Babuyan Claro Island.: KU 304796–98, 304800–05, 304810–13, 304815–16, 304821–24, 304828 304834–35, 304846, 304850–51; Camiguin Norte Island, Cagayan Province, Municipality of Calayan, Barangay Balatubat-Kauringan: KU 308023 (*Luperosaurus* *angliit* paratype); SNOMNH 47008.

*Luperosaurus* *palawanensis*: Philippines, Palawan Isl., Palawan Province, Malatgaw River, SE of Thumb Peak, about 3.5 km WNW of Iwahig: CAS 134207 (holotype); Thumb Peak, about 7 km NW of Iwahig: CAS 136740 (paratype).

*Gekko* [subgenus *Balawangekko*] *gulat* (formerly *Luperosaurus gulat*; Brown et al. 2012; Wood et al. 2020): Philippines, Palawan Island, Palawan Province, Municipality of Rizal, Barangay Ransang, Mt. Mantalingajan, locally known as "Gunob."; KU 320541.

**SUPPLEMENTARY TABLE**

**Table S1.** Summary of specimens corresponding to genetic samples included in the study.

| **Species** | **Voucher** | **Locality** | **ND2 gene**  **Genbank Accession** |
| --- | --- | --- | --- |
| *Lepidodactylus moestus* | USNM 521730 | Ngerur Island, Palau | JN019079 |
| *Lepidodactylus balioburius* | KU 314011 | Batanes Province | MG780754 |
| *Lepidodactylus lugubris* | KU 331653 | Albay Province | MG780755 |
| *Luperosaurus alvarezi* sp. nov. | UPLBMNH  4622 | Barangay Tampayan, Mt.Guiting-Guiting Natural Park, Sibuyan Island | PX262121 |
| *Luperosaurus alvarezi* sp. nov. | PNM 9866 | Barangay Tampayan, Mt.Guiting-Guiting Natural Park, Sibuyan Island | PX262122 |
| *Luperosaurus angliit* | KU 322189 | Barangay Zabali, Municipality of Baler, Aurora Province, Luzon Island, Philippines | PX262128 |
| *Luperosaurus angliit* | KU 322190 | Barangay Zabali, Municipality of Baler, Aurora Province, Luzon Island, Philippines | PX262129 |
| *Luperosaurus cumingii* | TNHC 61910 | Philippines, Luzon Island, Albay Province, Municipality of Tiwi, Mt. Malinao | JQ437902 |
| *Luperosaurus joloensis* | KU 314947 | Barangay Pasonaca, Zamboanga City, Mindanao Island, Philippines | JQ437900 |
| *Luperosaurus macgregori* | KU 308023 | Camiguin Norte Island | PX262124 |
| *Luperosaurus macgregori* | SNOMNH 47008 | Camiguin Norte Island | PX262123 |
| *Luperosaurus macgregori* | ACD 6022 | Calayan Island, Cagayan Province, Philippines | PX262125 |
| *Luperosaurus macgregori* | ACD 6023 | Calayan Island, Cagayan Province, Philippines | PX262127 |
| *Luperosaurus macgregori* | ACD 6024 | Calayan Island, Cagayan Province, Philippines | PX262126 |

**Note:** ACD = Arvin Diesmos field series, specimen deposited at the National Museum of the Philippines; KU = University of Kansas Natural History Museum; PNM = Philippine National Museum; TNHC = Texas Natural History Collections, University of Texas at Austin; USNM = United States National Museum; UPLB-MNH = University of the Philippines Los Baños Museum of Natural History; SNOMNH = Sam Noble Oklahoma Museum of Natural History.

**SUPPLEMENTARY FIGURES**

**Figure S1.** Comparison of the precloacal–femoral region in female and male Luperosaurus alvarezi sp. nov. The female (UPLBMNH-Z-NS 4622, collected from Mt. Guiting-Guiting, Romblon Province, Sibuyan Island, Philippines) shows indistinct or poorly defined precloacal–femoral pores, whereas the male (bottom; PNM 9866, collected from thte same locality exhibits clearly developed pores in the same anatomical position near the cloacal–femoral region.

**
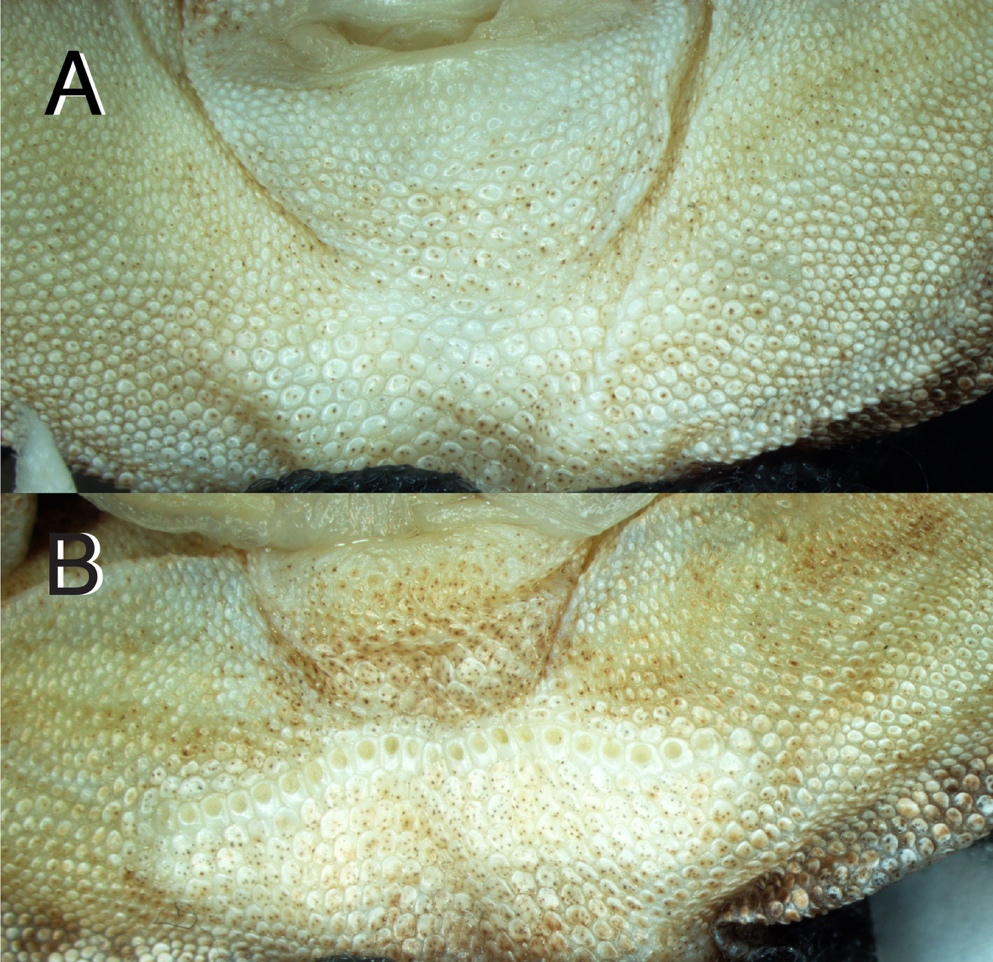
**

**Figure S2.** Female specimen of Luperosaurus alvarezi sp. nov. (UPLBMNH-Z-NS 4622) collected from Mt. Guiting-Guiting Natural Park. Shown in dorsal and ventral views to illustrate general morphology and preserved coloration.

**
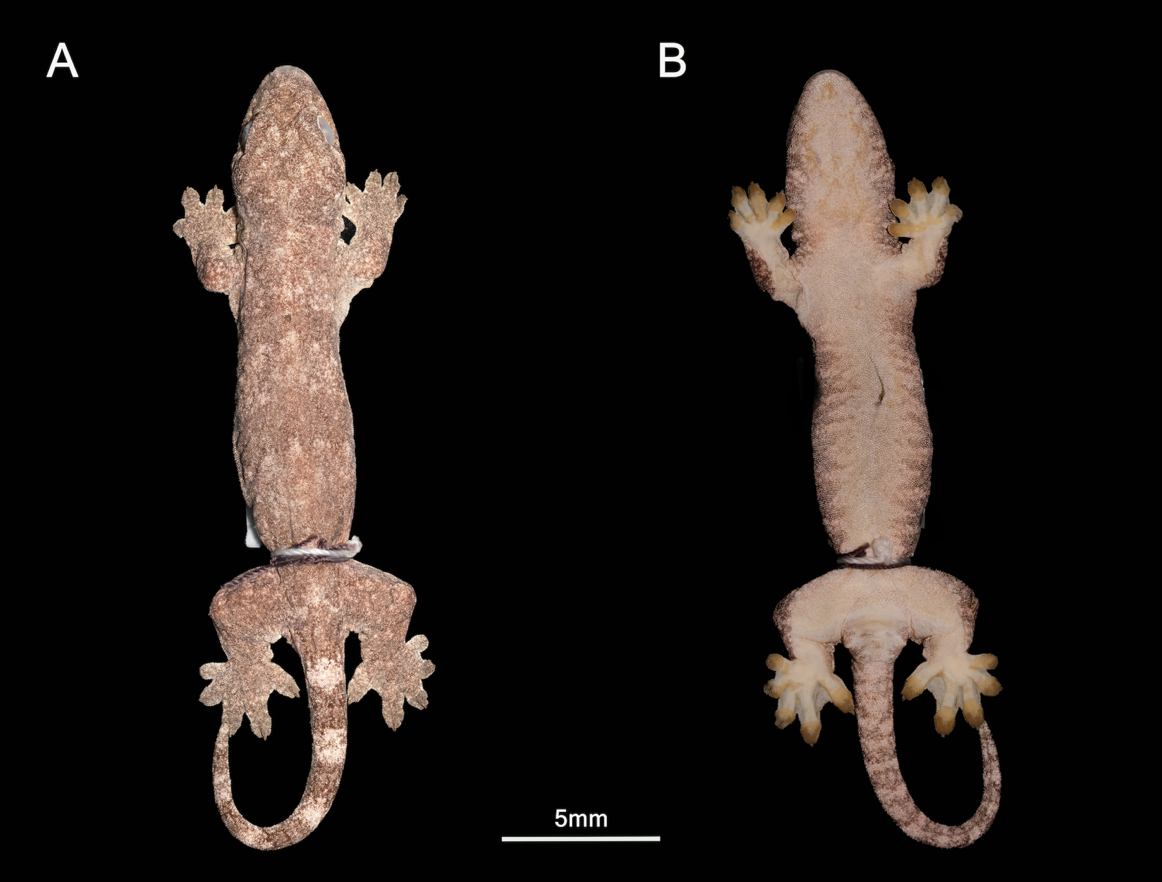
**

**Figure S3.** Bayesian inference tree estimated from 14 ND2 mitochondrial gene samples of *Luperosaurus*. Bayesian posterior probabilities are shown at nodes, with 1.00 indicating 100% support. The scale bar represents the number of substitutions per site.


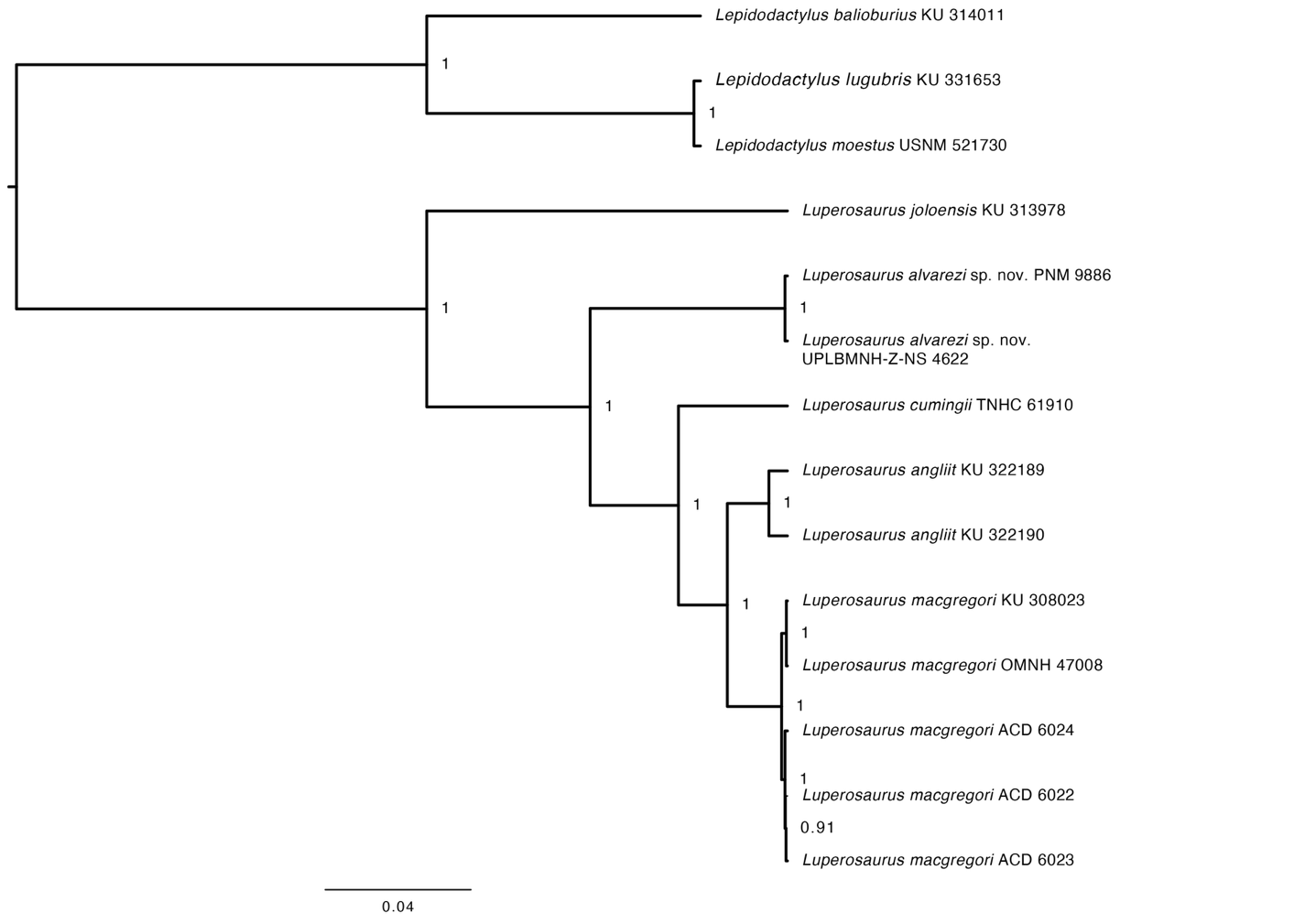

Supplement: Supplemental Information 4 [file peerj-14-20504-s004.docx]
